# Genome-wide association study of serum liver enzymes implicates diverse metabolic and liver pathology

**DOI:** 10.1038/s41467-020-20870-1

**Published:** 2021-02-05

**Authors:** Vincent L. Chen, Xiaomeng Du, Yanhua Chen, Annapurna Kuppa, Samuel K. Handelman, Rishel B. Vohnoutka, Patricia A. Peyser, Nicholette D. Palmer, Lawrence F. Bielak, Brian Halligan, Elizabeth K. Speliotes

**Affiliations:** 1 Division of Gastroenterology and Hepatology, University of Michigan Health System, Ann Arbor, MI USA; 2 Department of Computational Medicine and Bioinformatics, University of Michigan Medical School, Ann Arbor, MI USA; 3 Department of Epidemiology, University of Michigan School of Public Health, Ann Arbor, MI USA; 4 Department of Biochemistry, Wake Forest School of Medicine, Winston-Salem, NC USA

**Keywords:** Genome-wide association studies, Biomarkers, Liver diseases

## Abstract

Serum liver enzyme concentrations are the most frequently-used laboratory markers of liver disease, a major cause of mortality. We conduct a meta-analysis of genome-wide association studies of liver enzymes from UK BioBank and BioBank Japan. We identified 160 previously-unreported independent alanine aminotransferase, 190 aspartate aminotransferase, and 199 alkaline phosphatase genome-wide significant associations, with some affecting multiple different enzymes. Associated variants implicate genes that demonstrate diverse liver cell type expression and promote a range of metabolic and liver diseases. These findings provide insight into the pathophysiology of liver and other metabolic diseases that are associated with serum liver enzyme concentrations.

## Abstract (web summary)

Serum liver enzymes are used as markers of liver disease, their concentration influenced in part by genetic factors. Here the authors meta-analyse genome-wide association studies on the UK Biobank and BioBank Japan to evaluate the association of three liver enzymes with liver and other metabolic diseases.

## Introduction

Liver disease and its most serious complications of acute liver failure, cirrhosis, and liver cancer are leading and rising causes of mortality worldwide^1^. Liver enzymes such as alanine transaminase (ALT), aspartate transaminase (AST), and alkaline phosphatase (ALP) are the most commonly-used laboratory indicators of liver disease and are increased in most common liver diseases^2^. ALT and AST are traditionally considered markers of hepatocellular injury and ALP a marker of cholestasis. Liver enzyme elevations have been associated with greater liver-related mortality^3–5^ and are used in clinical practice guidelines to direct treatment decisions in liver disease^6–8^. Beyond liver disease, the presence of elevated liver enzymes has been associated with increased all-cause mortality and risk of cardiovascular disease and type 2 diabetes^3,4,9^.

Liver enzymes are partially genetically-determined, with heritability estimated around 0.5 based on twin studies^10,11^. Previously-published exome- or genome-wide association studies (GWAS) of genetic variants influencing liver enzyme concentrations identified loci in/near genes affecting bile acid transport, lipid/carbohydrate metabolism, glycobiology, and the immune system^12,13^. A more detailed understanding of genetic risk factors for liver enzymes may identify further mechanisms and pathways to yield deeper insight into the biology of liver diseases as well as identify those at high risk due to their genome. However, the existing studies on this topic utilized relatively small cohorts.

We aimed to further characterize the genetic architecture of liver disease with a GWAS to identify loci associated with ALT, AST, or ALP, using two cohorts with in total almost ten times as many participants than those of previously-published liver enzyme GWAS. In this work, we identify 378 loci associated with ALT, AST, and/or ALP at genome-wide significance. These loci are implicated in diverse metabolic and liver-related pathology, and are expressed in a wide range of cell types within the liver.

## Results

### Genome-wide association study and meta-analysis

**Fig. 1:**
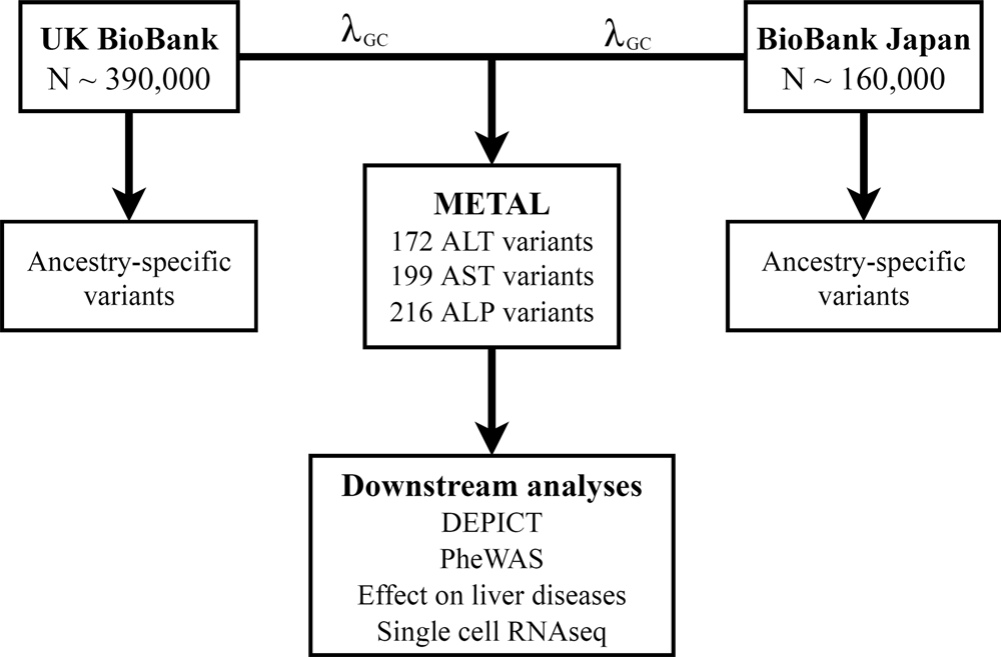
Study design. METAL is a software package that performs meta-analysis using genome-wide association study summary statistics. ALT, alanine aminotransferase. AST, aspartate aminotransferase. ALP, alkaline phosphatase. DEPICT, Data-driven Expression Prioritization Integration for Complex Traits. PheWAS, phenome-wide association study.

We performed a meta-analysis of variants affecting ALT, AST, or ALP using two large cohorts, UK BioBank (UKBB) and BioBank Japan (BBJ) (Fig. 1). In UKBB, we evaluated the additive effect of 23 million imputed autosomal genetic variants (with information score > 0.85) for effects on inverse normally-transformed serum ALT, AST, and ALP from over 389,565 individuals of European ancestry, adjusting for age, age^2^, sex, principal components 1–10, and relatedness using linear mixed modeling in SAIGE^14^. Basic demographic data and distributions of ALT, AST, and ALP in UKBB are shown in Supplementary Table 1. BBJ GWAS on serum ALT, AST, and ALP have been previously reported^15^ and included associations between ALT, AST, or ALP and 5,961,600 autosomal genetic variants from 162,255 Japanese individuals. Linkage disequilibrium (LD) score intercept values for ALT, AST, and ALP in UKBB were 1.26, 1.31, and 1.54, respectively, and in BBJ were 1.02, 1.01, and 1.06 suggesting that population structure in these datasets is well controlled (Supplementary Table 2). We conservatively performed full genomic inflation correction (lambda-GC) on each GWAS individually and performed meta-analysis using the sample size and p-value approach in METAL (a software package for GWAS meta-analyses) as previously reported^16^ and consistent with other trans-ethnic meta-analyses^17,18^. After meta-analysis, we removed triallelic variants, insertion-deletions, and variants with minor allele count < 0.001 in the combined cohort (UKBB plus BBJ), resulting in well-controlled genomic inflation for the overall meta-analysis with lambda-GC 1.03 for all three traits (Supplementary Table 2). We did not conduct additional genomic control for the meta-analysis. Genetic variants present in both studies with a combined *p*-value of <5 × 10^−8^ were considered replicated and used in downstream analysis. Quantile-quantile plots are shown in Supplementary Fig. 1. Regional association plots for genome-wide significant variants are available from the author upon request.

**Fig. 2:**
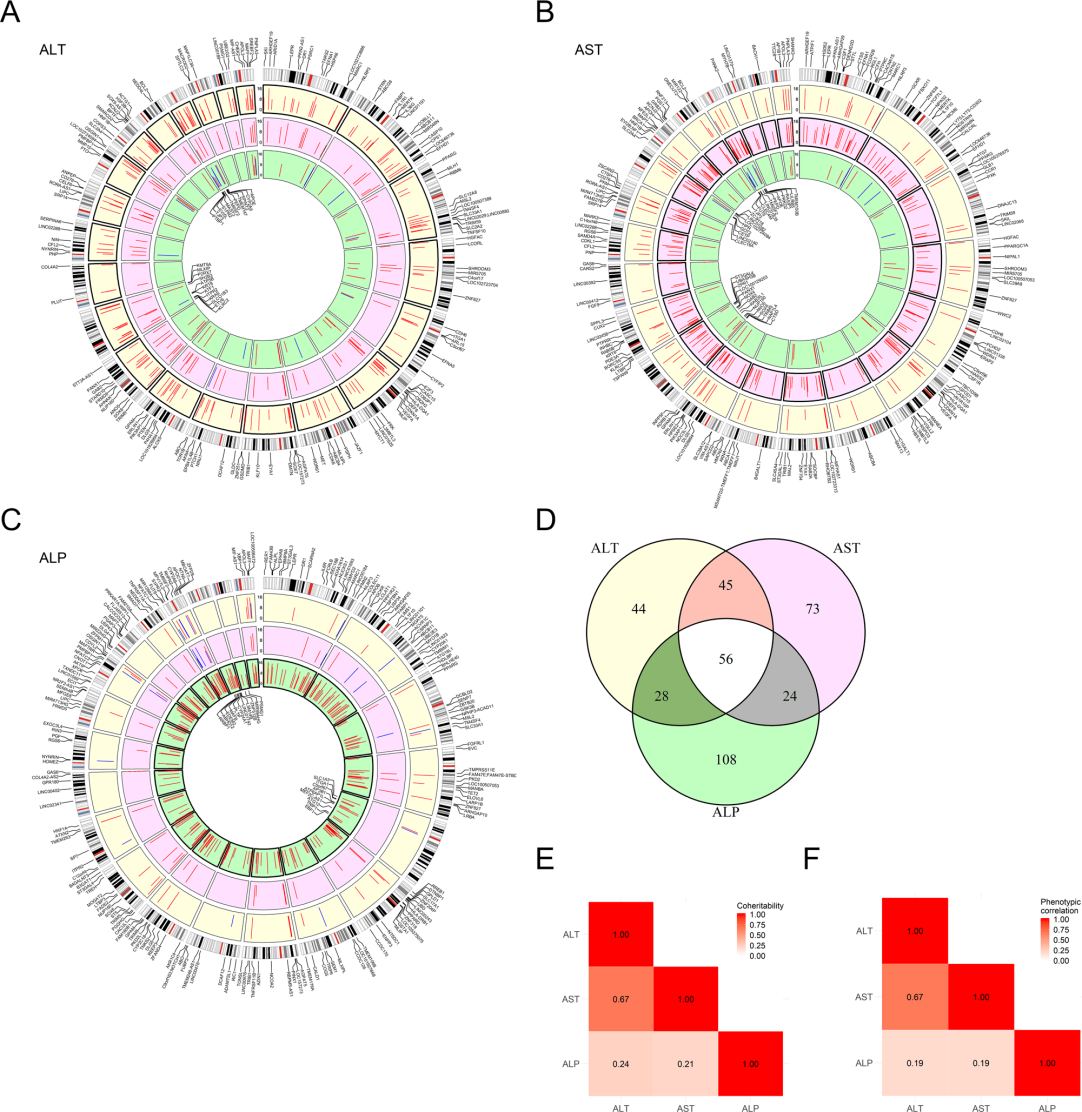
Genetic loci associated with liver enzymes. **A**–**C** Circos plots depicting variants associated at genome-wide significance (unadjusted *p* < 5 × 10^−8^) with **A** alanine transaminase (ALT), **B** aspartate transaminase (AST), and **C** alkaline phosphatase (ALP), and the associations of these variants with liver enzymes. Height of the bar represents –log_10_*p*-value of association. Outermost circles represent associations with ALT, middle circles with AST, and innermost circles with ALP. Plots are aligned to the target liver enzyme, i.e. ALT in **A**, AST in **B**, and ALP in **C**, so that the alleles that increase the target liver enzyme are used. (Note that for this reason different sub-figures may depict different alleles of the same variant.) Red color indicates that the allele associated with higher target liver enzyme increases that specific liver enzyme while blue indicates that that allele decreases the liver enzyme. Variants are labeled with their nearest gene or, for intergenic loci, the nearest upstream and downstream genes. *p*-values lower than 10^−16^ were rounded up to 10^−16^ for plotting purposes. A two-sided Z statistic was used to determine statistical significance. **D** overlap of loci across traits. **E** Co-heritability of ALT, AST, and ALP. **F** Phenotypic correlation of ALT, AST, and ALP.

We defined/identified 172 independent ALT, 199 AST, and 216 ALP loci after eliminating any SNPs within 1 Mb or LD (*R*^2^ > 0.01) of another genome-wide significant locus for the same trait (Fig. 2A–C; Supplementary Data 1–3). Of these loci, 160 ALT, 190 AST, and 199 ALP loci were novel (Supplementary Data 1–3). The overall list of variants constituted 378 distinct loci across the three traits after grouping variants that were within 1 Mb of another locus with lower p value for any trait (Fig. 2D, Supplementary Data 4). 153 variants had genome-wide significant associations with more than one trait (Fig. 2D). Overall, the direction of effect of alleles affecting both ALT and AST were more concordant with one another than either was with effects on ALP. Seventeen alleles were associated with increased ALT or AST but decreased ALP, or vice versa (Supplementary Data 1–3). In comparison, only one locus had alleles with opposite directions of effects on ALT and AST (two intronic variants of *ABO*). The coheritability between ALT and AST was 0.67, while that between ALT and ALP was 0.24 and between AST and ALP 0.21 (Fig. 2E). The phenotypic Pearson correlation was 0.67 between ALT and AST, and 0.19 between either ALT and ALP or AST and ALP (Fig. 2F).

Statistically-significant heterogeneity (*p*_het_ < 0.05 after Bonferroni correction) was found between UKBB and BBJ for only 20 ALT-, 14 AST-, and 24 ALP-increasing alleles (Supplementary Tables 3–5). Of note, the direction of effect was concordant between UKBB and BBJ for nearly all of these alleles (19/20 for ALT, 13/14 for AST, and 21/24 for ALP), suggesting overall congruency of association across the populations.

Our primary analysis focused on variants common across ancestries, but we also conducted a secondary analysis on ancestry-specific variants. We identified 144, 181, and 319 genetic variants associated at genome-wide significance with ALT, AST, or ALP, respectively, in UKBB but not in the meta-analysis (Supplementary Data 5–7). There were also two and five genetic variants associated with ALT and AST at genome-wide significant levels, respectively, in BBJ but not the meta-analysis (Supplementary Tables 6–7); there were no genome-wide significant BBJ-specific ALP genetic variants. UKBB-specific variants included exonic variants in *SERPINA1* (Supplementary Data 7) and *HFE* (Supplementary Data 5) responsible for the alpha-1 antitrypsin Z phenotype and hereditary hemochromatosis, respectively^19,20^, with a disease causing allele frequency of 0.02 and 0.08, respectively, in UKBB and are not present (monomorphic for the major allele) in BBJ, highlighting one reason certain liver diseases are more prevalent in certain populations. Other reasons for some genetic variants not being present in BBJ include lower sample size or poor genotyping or imputation quality. 71% of associations for ALT, 81% of associations for AST, and 70% of associations for ALP in BBJ are directionally consistent with effects in UKBB when effects in both are seen suggesting that 32, 59, and 54 of these associations are likely to represent true associations for these traits (sign test *p* < 0.0004).

We found overall congruency of effect of most the alleles in men and women with most not having significant p values for heterogeneity across sexes in UKBB. We identified 7 ALT, 5 AST, and 8 ALP alleles with Bonferroni-adjusted significant heterogeneity of effect between men and women (Supplementary Tables 8–10) and report the effects in men and women separately for these. None of these alleles had opposite directions of effect in men and in women, and only three variants were significantly associated with a liver enzyme in one sex but not the other (Supplementary Table 9).

### Gene, pathway, and tissue analyses

**Fig. 3:**
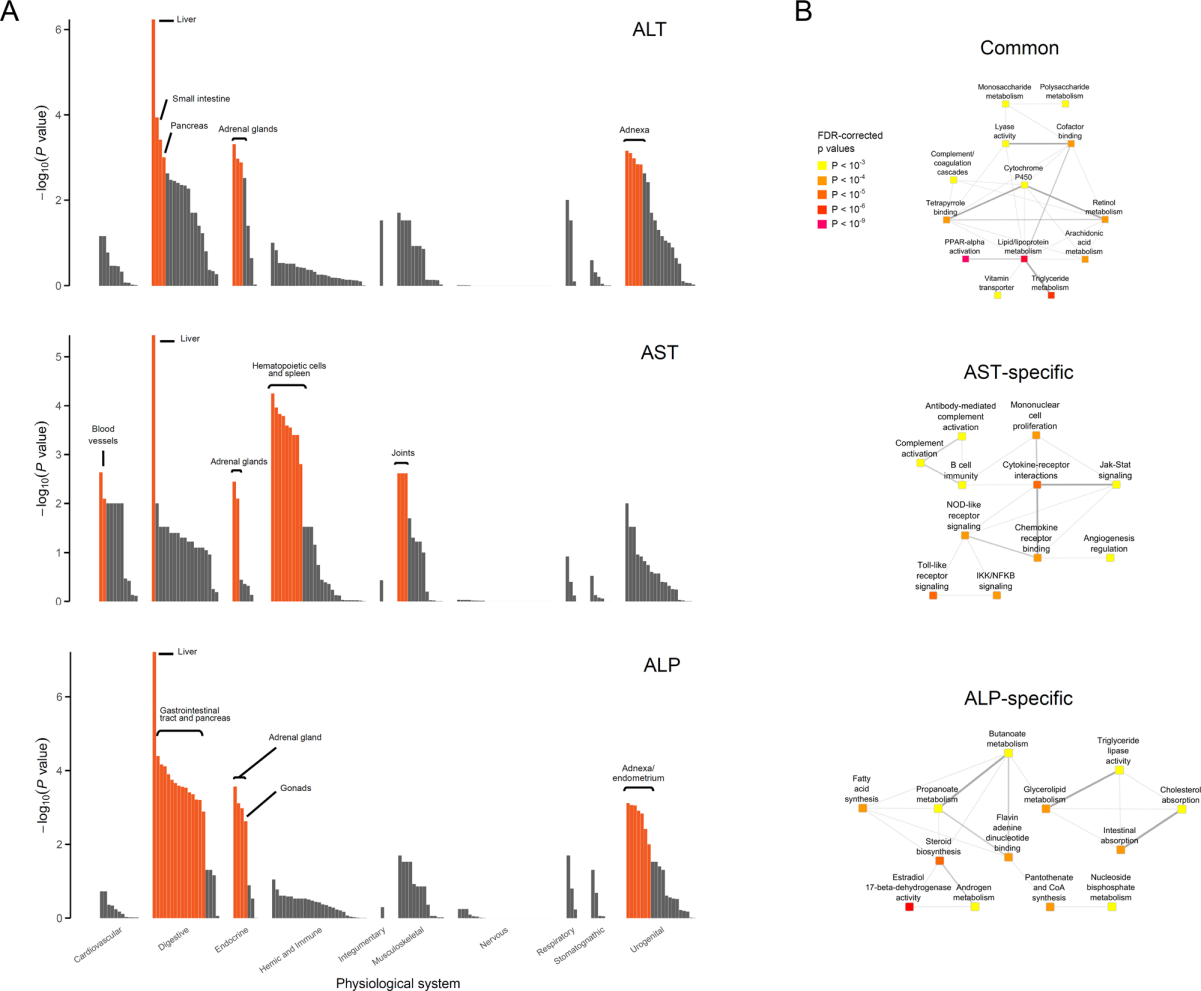
DEPICT analysis of liver enzyme-associated variants. **A** Tissue enrichment of alanine transaminase (ALT), aspartate transaminase (AST), or alkaline-phosphatase (ALP) associated genetic variants. Height of the bar represents –log_10_p-value. Orange shading represents statistical significance at false discovery rate (FDR) < 0.05. **B** Network plots depicting gene sets enriched in all three sets of ALT-, AST-, and ALP-associated variants (“Common”), in gene sets enriched only among AST-associated variants (“AST-specific”), and in gene sets enriched only among ALP-associated variants (“ALP-specific”).

We used DEPICT^21^, a program that uses GWAS-prioritized genes and gene co-expression patterns across cells and tissues, to identify tissues, pathways, and genes (using an FDR < 0.05) that are enriched for associations with a trait (Fig. 3; Supplementary Data 8–13). For ALT, AST, and ALP-prioritized genes, liver was consistently the most enriched tissue. In addition to liver, ALT-prioritized genes were enriched in small intestine, pancreas, adrenal, and adnexa (Fig. 3A, Supplementary Data 8), while AST-prioritized genes were enriched in hematopoietic cells and spleen, joints, adrenal glands, and blood vessels (Fig. 3A, Supplementary Data 9). ALP-prioritized genes were enriched in the entire gastrointestinal tract and pancreas, adrenal glands, and primary and secondary sexual organs (Fig. 3A; Supplementary Data 10). The union of the gene sets enriched among ALT-, AST-, and ALP-increasing alleles included pathways related to metabolism of lipids/lipoproteins, carbohydrates, retinol, and arachidonic acid, as well as *PPARA* activation, retinoid X receptor, cytochrome P450, and complement/coagulation cascades (Fig. 3B; Supplementary Data 11–13). AST-specific gene sets included inflammatory gene biology: NOD-like, Toll-like and chemokine receptor signaling, NFkB signaling, JAK-STAT signaling, and B cell biology (Fig. 3B; Supplementary Data 12). ALP-specific gene sets included different aspects of metabolism such as sex hormone activity/metabolism, cholesterol absorption, and glycerolipid metabolism (Fig. 3B), and ALT-specific gene sets included ABC transporters, metal ion SLC transporters, and hydrolase activity (Supplementary Data 11).

### Pleiotropism analyses: diagnoses

**Fig. 4:**
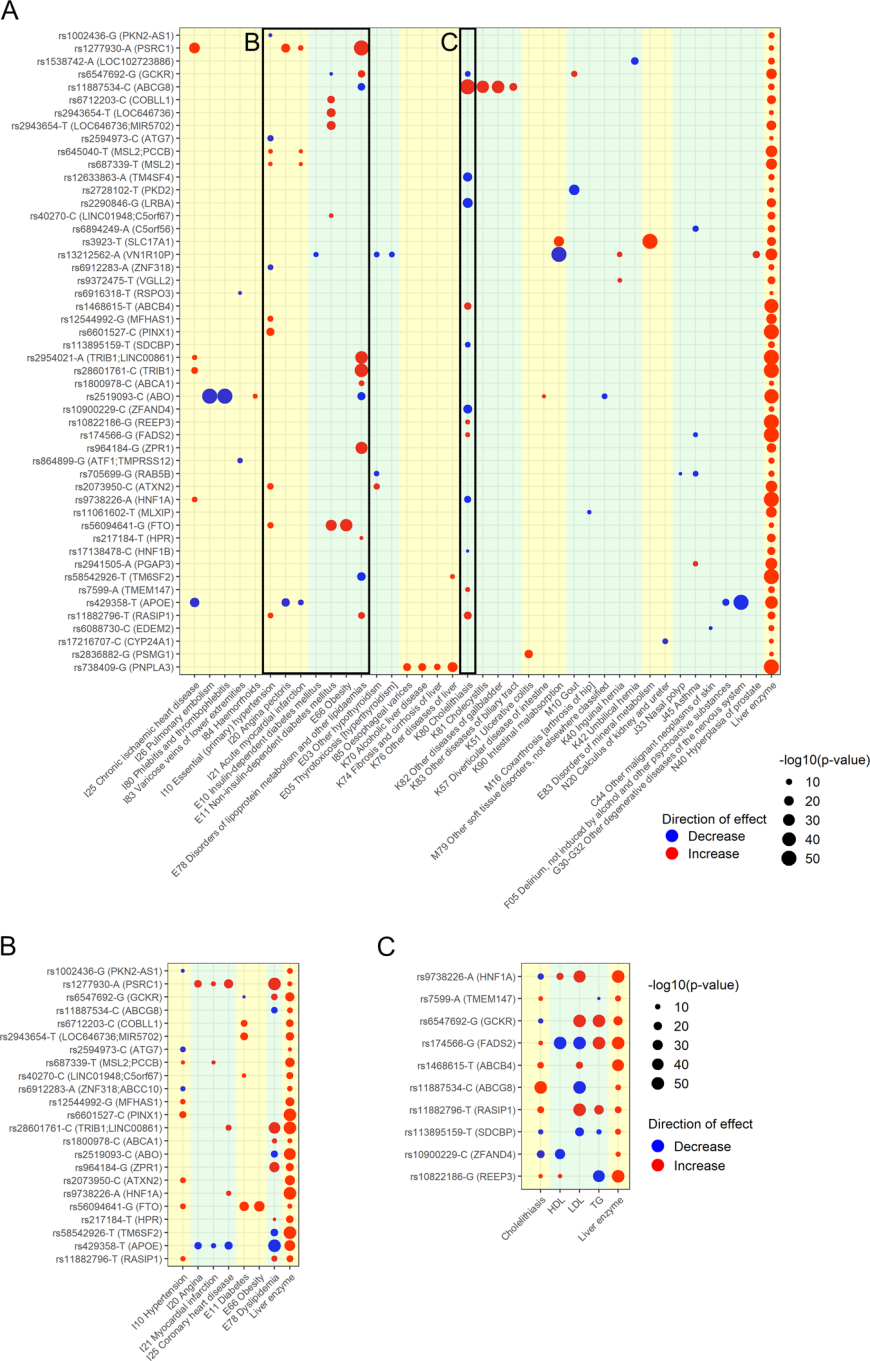
Phenome-wide association study of liver enzyme associated variants. **A** All associations between liver enzyme-associated variants and diseases. **B** Associations between liver enzyme-associated variants and metabolic diseases. **C** Associations between liver enzyme-associated variants, cholelithiasis, and lipid traits, namely high-density lipoprotein (HDL), low-density lipoprotein (LDL), and triglycerides (TG). For all panels, only genome wide-significant associations (unadjusted *p* < 5 ×10^−8^) are shown. Red indicates that the liver enzyme-increasing allele increases the disease/trait, while blue indicates that it decreases it. Larger circles indicate lower *p*-value. Background shade denotes organ system associated with the diseases. Data for **A** and **B** are from^22^ while data on HDL, LDL, and TG are based on genome-wide association studies in UK BioBank. **B** and **C** use the same scale while **A** uses a different scale. When more than one variant at a given locus (i.e. <1 MB apart) was associated with different liver enzymes, only the variant with the most significant association with a liver enzyme was included. (For example, if one variant was associated with alanine aminotransferase with *p* = 4 × 10^−8^ and another nearby variant was associated with aspartate aminotransferase with *p* = 2 × 10^−8^, the latter would be used.) Note that rs5854926 (*TM6SF2*) had opposite effects on alanine aminotransferase and alkaline phosphatase and on this plot is aligned to the alanine aminotransferase-increasing allele. A two-sided Z statistic was used.

Next, we carried out phenome-wide association studies (PheWAS) of ALT-, AST-, and ALP-increasing alleles with International Classification of Diseases (ICD)-based diseases using previously-published PheWAS data^22^. We found a number of significant allele-disease associations (*p* < 5 × 10^−8^) between ALT-, AST- or ALP-increasing alleles and 38 human diseases (Fig. 4; Supplementary Tables 11–13). Notable findings were that a number of alleles were associated with metabolic diseases and cholelithiasis. 23 alleles associated with metabolic traits including hypertension, dyslipidemia, diabetes, and coronary artery disease (Fig. 4B). 13 alleles associated with variations in risk of cholelithiasis (Fig. 4C). Several alleles associated with other traits of interest: rs2836882 near *PSMG1* associated with ulcerative colitis, multiple SNPs including rs3923 near *SLC17A1* associated with disorders in mineral metabolism, and rs17216707 near *CYP24A1* associated with nephrolithiasis.

### Pleiotropism analyses: traits

Unbiased PheWAS analysis identified numerous genetic variants associated with metabolic diseases and cholelithiasis, which is most frequently caused by precipitation of cholesterol in the gallbladder. Given this, we further investigated the effects of liver enzyme-increasing alleles on metabolic traits using more powered continuous versions of these traits (Supplementary Fig. 2; Supplementary Data 14–16). Many liver enzyme-associated alleles showed variation in high-density lipoprotein (HDL; *n* = 104), low-density lipoprotein (LDL; *n* = 107), or triglycerides (TG; *n* = 104) (Supplementary Fig. 2). Anthropometric traits were also frequently associated: 34 genetic variants associated with body mass index (BMI), 20 with waist circumference, and 46 with waist-hip ratio adjusted for BMI (Supplementary Fig. 2, Supplementary Data 14–16). Further, all genetic variants associated with variation in risk of cholelithiasis also associated with variation in serum lipid concentrations (Fig. 4C). We also evaluated associations between serum metabolites and liver enzyme-associated alleles based on previously published summary statistics^23^; we found diverse effects (Supplementary Fig. 3; Supplementary Data 17–19). For instance, an ALT-increasing allele in *TM6SF2* was associated with decreased intermediate-, low-, and very low-density lipoproteins, while an ALT-increasing allele near *MLXIPL* associated with a decrease in only very low-density lipoproteins, and another ALT-increasing *APOE* allele associated with decreased very low- and LDLs but increased HDL concentrations (Supplementary Fig. 3). These findings suggest that distinct metabolic and anthropometric alterations may be important mechanisms by which liver enzyme-increasing alleles cause liver damage.

### Pleiotropism analyses: causal inference

Given that liver enzyme-increasing alleles also were associated with multiple metabolic traits in UKBB, we sought to determine direction of effect: specifically, if elevated liver enzymes (presumably reflecting underlying liver disease) resulted in altered metabolism, or vice versa. We did this in two ways. First, we evaluated the variance explained for their respective traits of mutations in the genes coding for the liver enzymes themselves that were most strongly-associated with their respective liver enzyme in UKBB. We chose alleles in *ALPL* (rs1256330-T, beta = 0.11, *p* = 1.59 × 10^−298^, variance explained 0.005 for ALP), *GPT* (rs141505249-C, beta = 1.6, *p* < 1 × 10^−300^, variance explained 0.025 for ALT), *GOT1* (rs146049867-T, beta = 0.69, *p* = 1.41 × 10^−65^, variance explained 0.0006 for AST), and *GOT2* (rs11076256-T, beta = 0.08, *p* = 2.07 × 10^−78^, variance explained 0.0006 for AST). The coding variants in *ALPL, GPT*, *GOT1*, *GOT2* did not have effects on any metabolic trait despite >90% power to detect variance explained >0.0004 on all of these traits (Supplementary Fig. 2). This suggests that ALP, ALT, and AST are not causally related to development of the metabolic changes tested. Second, we evaluated causal relationships between ALT, AST, or ALP and continuous metabolic traits in UKBB using latent causal variable analysis^24^. We chose these metabolic traits because they were both strongly associated with liver enzyme-altering variants and highly statistically powered (Methods). Only traits for which there was evidence of both non-zero genetic causality proportion and of non-zero genetic correlation (rho) with a liver enzyme were considered causal. We found that genetic predisposition for decreased high-density lipoprotein and increased BMI also causally increased ALT (Supplementary Table 14). There was insufficient evidence that genetic predisposition for any of the metabolic traits causally increased AST (Supplementary Table 15). ALP did not demonstrate high heritability (Z score < 7) so causal inference analysis involving ALP was not interpreted due to possible inflated p values (Supplementary Table 16). There was no significant evidence for causality of ALT, AST, or ALP with any metabolic traits. These analyses suggest that liver enzyme elevations themselves do not cause metabolic diseases, but instead are the result of metabolic disease such as obesity and dyslipidemia.

### Pleiotropism analyses: human liver disease

**Fig. 5:**
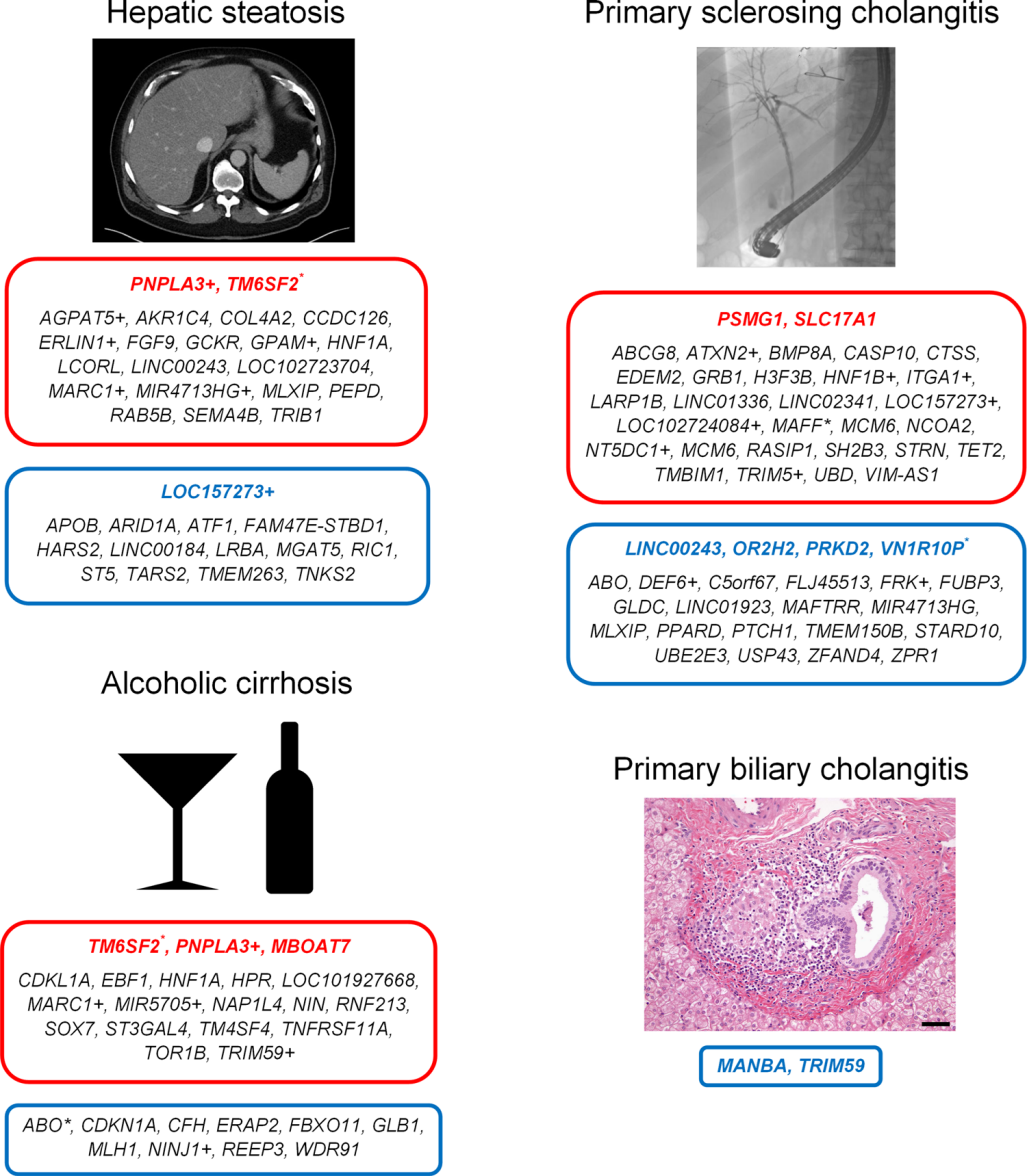
Associations of liver enzyme-increasing genetic variants on liver diseases. The nearest gene to each variant is depicted. Red text indicates that the liver enzyme-increasing allele increases risk of that disease, while blue text indicates that it lowers risk of that disease (Bonferroni-corrected). Black text indicates a trend toward a significant effect (*p* < 0.05). When two or three variants <1 MB apart are associated with different liver enzymes are also associated with a liver trait, this was considered a single genetic locus and the variant with the lowest *p*-value for its respective liver enzyme was used. These variants are highlighted with * indicating that the locus had opposite directions of association on the liver enzymes, or with + indicating that the locus had the same direction of association on the liver enzymes. Hepatic steatosis is defined as negative computed tomography-measured liver attenuation, so that an allele that is associated with increased liver attenuation would be associated with decreased hepatic steatosis, and vice versa. Scale bar length is 50 micrometers. The primary biliary cholangitis micrograph is courtesy of Dr. Henry Appelman at University of Michigan.

Having found using PheWAS analysis that liver enzyme-increasing variants associated with clinically-relevant disease using ICD codes, we sought to determine how they were associated with human liver diseases. We evaluated their associations with computed tomography-measured liver attenuation (lower liver attenuation implies more hepatic steatosis)^25^, alcoholic cirrhosis^26^, primary biliary cholangitis^27^, and primary sclerosing cholangitis^28^ using previously published summary statistics. Disease associations are shown in Fig. 5, Supplementary Table 17, and Supplementary Data 20–28. A *p*-value Bonferroni-corrected for 378 independent liver enzyme loci was used to determine significance (*p* < 1.3 × 10^−4^); alleles trending toward significance (*p* < 0.05) were also reported separately. Numerous liver enzyme-associated alleles were also associated with liver diseases (Fig. 5). While some of these associations were known, such as with *PNPLA3* and *TM6SF2* with hepatic steatosis and *HLA* variants with primary sclerosing cholangitis, many were novel, such as between primary sclerosing cholangitis and a missense mutation in *SLC17A1*, which is involved in phosphate and urate transport. This finding suggests that some liver enzyme-associated alleles predispose to liver disease and not just circulating marker levels.

**Fig. 6:**
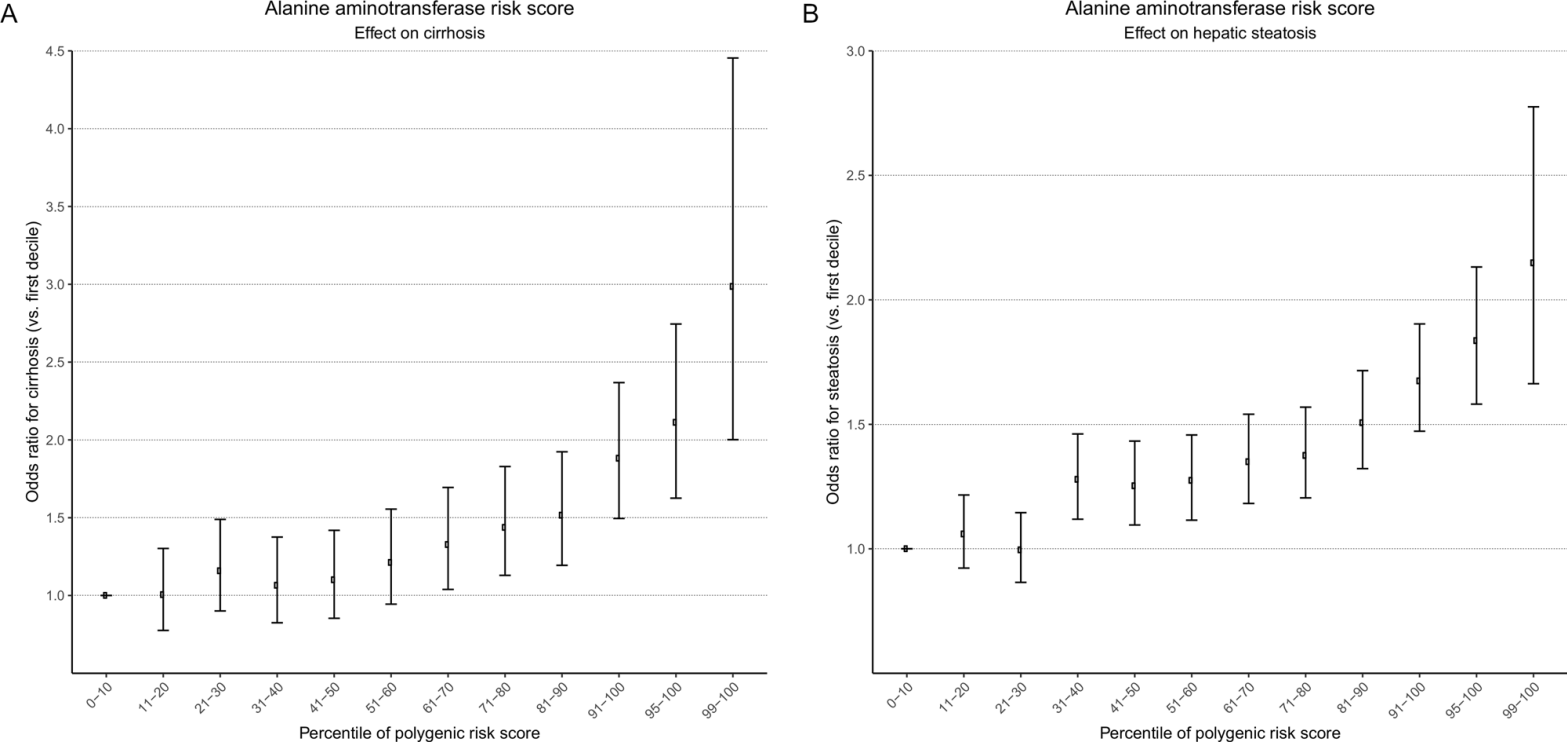
Associations between alanine aminotransferase polygenic risk score and cirrhosis and steatosis. **A**, **B** Association between percentile of alanine aminotransferase polygenic risk score on **A** cirrhosis or **B** steatosis. All results are depicted as odds ratios for cirrhosis or steatosis relative to individuals in the 0-10th percentile of polygenic risk score, adjusted for sex, age, age^2^, and principal components 1–10. Error bars represent 95% confidence intervals. *N* = 51,550.

As further evidence that the variants associated with liver enzyme concentrations have effects on clinically-relevant liver disease, we evaluated their effects on steatosis and all-cause cirrhosis using an independent cohort, Michigan Genomics Initiative (MGI). We created polygenic risk scores (PRSs) for ALT, AST, and ALP based on a sum of dosage of independent variants associated with them at genome-wide significance (5 × 10^−8^; Supplementary Data 1–3), weighted based on beta values in UKBB for their respective traits. Higher PRS was strongly associated with ALT, AST, and ALP concentration in MGI (*N* = 35, 730), with beta values for each rank unit of the inverse-normally-transformed ALT, AST, or ALP PRS on ALT, AST, or ALP of 0.15 (95% CI 0.14–0.16), 0.16 (0.15–0.17), and 0.23 (0.22–0.24), respectively. Each rank unit of ALT PRS was associated with an odds ratio (OR) of 1.23 (1.17–1.30) for cirrhosis and 1.17 (1.14–1.21) for steatosis (Supplementary Table 18) in MGI. Higher AST PRS was also associated with increased odds of both cirrhosis and steatosis, though this association was weaker than that of ALT, while the ALP PRS was not significantly associated with either cirrhosis or steatosis (Supplementary Table 18). Compared to those with the bottom decile of ALT PRS, individuals in the top 10%, 5%, and 1% of ALT PRS had OR 1.88, 2.11, and 2.99 for cirrhosis, and OR 1.67, 1.84, and 2.15 for hepatic steatosis (Fig. 6). A similar trend was seen for AST PRS percentile and cirrhosis and steatosis (Supplementary Fig. 4).

### Mendelian diseases

Next, we investigated associations between non-synonymous mutations in LD with the liver enzyme-associated variants and Mendelian diseases using the Online Mendelian Inheritance in Man catalog (Table 1, Supplementary Data 29, Methods). One of the ALT-increasing allele is a coding *ABCG8* variant previously associated with the Mendelian disease sitosterolemia (Table 1). Other potentially deleterious missense mutations in LD with ALT-increasing alleles were found in *ANO5*, which is associated with Miyoshi muscular dystrophy; the breast/ovarian cancer susceptibility gene *BRCA1*; and *LRBA*, associated with common variable immunodeficiency. These LD exonic alleles were associated with abnormal liver enzymes even after adjustment for previously-reported Mendelian disease variants and constitute new physiology-altering if not disease-causing variants.

**Table 1:** Genetic variants associated with both liver enzyme levels and Mendelian disease genes.

Disease	Mendelian disease gene	Liver enzyme lead SNP	Exonic SNP	Exonic SNP deleteriousness			
				CP	Sift	HD	HV
*Sitosterolemia*	*ABCG8*	*rs11887534*	*rs11887534*	*22.7*	*D*	*P*	*P*
Sitosterolemia	ABCG5	rs11887534	rs6756629	23.0	D	D	P
**Gnathodiaphyseal dysplasia; Miyoshi muscular dystrophy 3**	**ANO5**	**rs7481951**	**rs7481951**	**12.1**	**T**	**B**	**B**
Carbamoyl phosphate synthetase I deficiency	CPS1	rs1047891	rs1047891	22.1	T	B	B
Lipoid proteinosis of Urbach and Wiethe	ECM1	rs1815544	rs3737240; rs13294	0.1; 9.4	T; T	D; D	P; B
Spastic paraplegia	ERLIN1	rs2862954	rs2862954	13.3	T	B	B
Deafness	S1PR2	rs10409243	rs2116942	NA	NA	NA	NA
Ceroid lipofuscinosis, neuronal, 10	CTSD	rs11555039	rs17571	1.5	T	B	B
**Breast-ovarian cancer, familial; Fanconi anemia, complementation group S**	**BRCA1**	**rs8176279**	**rs1799966**	**0.5**	**T**	**P**	**B**
Retinitis pigmentosa	MERTK	rs6541998	rs7604639; rs2230515	0.0	T	B	B
Malignant hyperthermia;Hypokalemic periodic paralysis; Thyrotoxic periodic paralysis	CACNA1S	rs3850625	rs3850625	34.0	D	P	B
Brain small vessel disease	COL4A2	rs9521787	rs7990383	0.0	T	B	B
Ellis-van Creveld syndrome; Weyers acrofacial dysostosis	EVC	rs2279252	rs2279252	23.0	T	P	B
Immunodeficiency, common variable	IRF2BP2	rs599633	rs7545855	0.0	T	B	B
Immunodeficiency, common variable, 8, with autoimmunity	LRBA	rs2290846	rs2290846; rs3749574	25.9; 19.9	T	B	B
Mannosidosis, beta B, lysosomal	MANBA	rs179195	rs2866413	6.5	T	B	B
Encephalitis/encephalopathy, mild, with reversible myelin vacuolization; Cardiac-urogenital syndrome	MYRF	rs174566	rs174535	NA	NA	NA	NA
Lethal congenital contracture syndrome; Nevus comedonicus	NEK9	rs4903273	rs10146482	18.7	T	B	B
**Acrodermatitis enteropathica, zinc-deficiency type**	**SLC39A4**	**rs13273326**	**rs2280838; rs2280839**	**8.0; 5.7**	**T; D**	**B; B**	**B; B**
Paget disease of bone, juvenile-onset	TNFRSF11B	rs11573824	rs2073618	16.0	T	B	B
Trehalase deficiency	TREH	rs12225548	rs2276065	0.0	T	B	B
Combined oxidative phosphorylation deficiency	VARS2	rs9501030	rs9394021	27.5	D	D	D
Cerebellar ataxia, mental retardation, and dysequilibrium syndrome; Hydrocephalus, congenital, with brain anomalies	WDR81	rs11078597	rs8077638	NA	NA	NA	NA

*SNP* single nucleotide polymorphism, *CP* Combined Annotation-Dependent Depletion-Phred, HD HumDiv (PolyPhen), *HV* HumVar (PolyPhen), *NA* not available.

Bold denotes that the liver enzyme-affecting variant influences liver enzymes independently of previously-reported Mendelian disease-causing variants. Italics denotes that the liver enzyme-affecting variant is the same as a previously-reported Mendelian disease-causing variant.

### Single cell transcriptomics

**Fig. 7:**
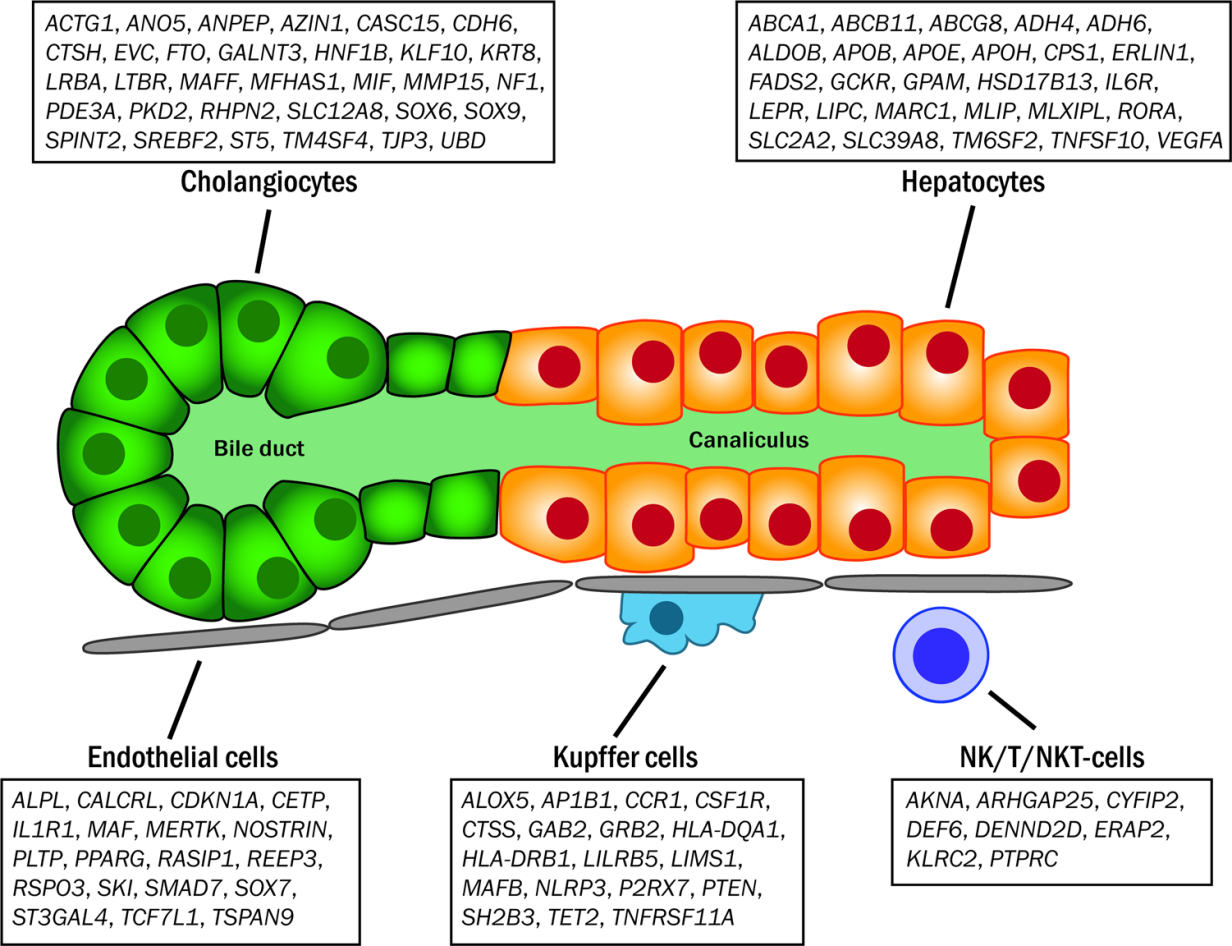
Cell type-specific expression of genes nearest to selected liver enzyme-associated genetic variants. Gene names appear in the boxes corresponding to the cell type in which they are specifically expressed.

Finally, we used recently-reported human single cell RNA sequencing data from human liver to investigate cell-specific expression of the genes nearest to the liver enzyme-altering variants (Fig. 7; Supplementary Data 30–31)^29^. Because the RNA sequencing protocol enriched for polyadenylated RNA, we also investigated expression of the nearest coding genes to the liver enzyme-altering variants. 92% (693/752) of these genes were present in the RNA sequencing dataset. We then identified genes that were expressed at higher levels in one of five cell populations: hepatocytes, cholangiocytes, Kupffer cells, endothelial cells, or natural killer/T/ natural killer-T cells (Methods), compared to other cell types. In total, 85 genes were more highly expressed in hepatocytes, 118 in cholangiocytes, 40 in Kupffer cells, 37 in endothelial cells, and 15 in natural killer/T/ natural killer-T cells (Supplementary Data 30). Hepatocyte-specific genes were typically those associated with liver physiological processes such as small molecule and cholesterol transport (*ABCA1*, *ABCG8*, *SLC39A8*), lipid biology (*APOB*, *APOE*, *FADS2*, *LIPC*, *GPAM*), and carbohydrate metabolism (*MLXIPL*, *GCKR*). In contrast, genes associated with cholangiocytes were more associated with cancer (*CDH6*, *ST5*), epithelial-mesenchymal transition (*LRBA*, *TJP3*), and stem cell-related phenotypes (*RHPN2*, *HNF1B*), consistent with prior reports that these cells form a class combined with EpCAM-expressing progenitor cells^29^. Endothelial cells expressed a variety of genes involved in lipid transport and metabolism (*CETP*, *PPARG*, *PLTP*) and inflammation/adhesion (*NOSTRIN*, *IL1R1*). As expected, Kupffer cell- and NK/T/NKT cell-specific genes were primarily involved in immunity (*DEF6*, *HLA-DRB1*, *NLRP3*).

## Discussion

We identified 378 independent loci associated with serum liver enzyme concentrations, of which 160 ALT, 190 AST, and 199 ALP were novel. These loci have diverse pleiotropic effects on human disease, including liver disease, and prioritized genes based on these loci are expressed in all major cell populations in the liver and diverse tissues outside of liver. These findings greatly enhance our understanding of the genetic basis of human liver disease.

Each liver enzyme has a distinct genetic architecture: only 40% of variants associated with more than one liver enzyme at genome-wide significance. ALT and AST were more coheritable than were either ALT and ALP or AST and ALP. Similarly, while only one allele associated with increased ALT and decreased AST at genome-wide significance, there were many more instances where alleles associated with increased ALT or AST were associated with decreased ALP. This is consistent with the idea that elevations in ALT and AST reflect hepatocellular disease while ALP reflects cholestasis^2^. PheWAS and targeted analysis of liver diseases similarly showed distinctions between the different liver enzymes. For example, ALP is in addition to its effects in the liver also important in bone maintenance and intestinal barrier function^30,31^. Two alleles associated with increased ALP, rs3923-T (*SLC17A1* missense mutation) and rs764284-G (near *CYP24A1*), were associated with mineral metabolism disorders and intestinal malabsorption. SLC17A1 is, among other things, a sodium-phosphate cotransporter that increases phosphate reabsorption in the proximal tubule, which suggests that rs3923-T could affect ALP concentration via phosphate/bone metabolism. *CYP24A1* is the primary catabolic enzyme for 1,25-dihydroxyvitamin D and 25-hydroxyvitamin D, and modulation of *CYP24A1* may also cause bone disease and contribute to ALP elevations. Similarly, the ALP-associated allele rs2836882-G (near *PSMG1*, a proteasome assembly chaperone) is associated with ulcerative colitis, which is itself strongly associated with the cholestatic inflammatory liver disease primary sclerosing cholangitis resulting in elevations in alkaline phosphatase^32^. We verified that rs2836882-G is also associated with primary sclerosing cholangitis at genome-wide significance (Fig. 5), suggesting that this association is the most likely mechanism underlying this variant’s effect on ALP. Thus, PheWAS may assist in elucidating the disease biology underlying liver enzyme elevation and identifying patterns of associations that mark subtypes of disease.

We found on PheWAS that genetic variants in/near the genes coding for ALT (*GPT*), AST (*GOT1*/*GOT2*), and ALP (*ALPL*) did not themselves associate with liver diseases or other diagnoses suggesting that the liver enzymes are likely not themselves pathogenic. Some genetic variants associated with these enzyme levels, however, do associate with common liver diseases. We validated several previously-reported variant-nonalcoholic fatty liver disease associations (*PNPLA3, TM6SF2, GCKR*), and also identified new associations between variants in/near *APOB* and hepatic steatosis and between primary sclerosing cholangitis and a variant near *VN1R10P*, which is involved in regulation of innate and adaptive immunity including in diseases such as rheumatoid arthritis and Sjogren syndrome. Other variants likely mark subtle forms of glycogen storage disease (*LOC157273*)^33^. Still other variants define known Mendelian syndromes that affect the liver such as sitosterolemia or define coding variants with novel human effects. A major reason we did not identify more associations between liver enzyme-altering alleles and liver diseases is likely power, as UKBB and BBJ are collectively >20 times larger than any of the publicly-available cohorts of genotyped participants with the specific liver disease phenotypes used in this study. Liver enzyme changes may therefore be a more statistically-powered alternative to identify disease alleles in population studies.

We identified a number of ancestry-specific variants affecting liver enzymes, with >100 UKBB-specific ALT-, > 100 AST-, and >300 ALP-associated variants, and several BBJ-specific ALT- or AST-associated variants. Allele frequency differences are one reason genetic variants had effects in one but not the other ancestry. Two prime examples are the variants in *SERPINA1* and *HFE* responsible for alpha-1 antitrypsin deficiency and hereditary hemochromatosis that are relatively common in individuals of European ancestry but rare in East Asians. When alleles were present in both ancestries we saw an enrichment for directionally congruent effects across the ancestries suggesting that many of these variants are likely to be real for associating with liver function tests across ancestries and will become significant in future analyses with larger sample sizes. Some ancestry-specific loci have plausible biologic relevance in roles such as lipid metabolism (e.g., UKBB-specific AST variant in *APOM*), retinoid metabolism (BBJ-specific ALP variant near *NCOA2*), or inflammation (BBJ-specific ALP variant near *TNFSF11*). As individual-level data from BBJ are not available, we were not able to determine whether variants missing from BBJ were excluded due to low minor allele frequency (<0.01) or poor imputation/genotyping quality^34^. Further investigation will be required to determine the importance of these variants in human health.

Some clinically-relevant findings in this study include pleiotropic effects of alleles associated with liver enzyme levels that may have implications both for therapeutic drug targeting and in identifying mechanisms of disease. Several variants associate with both liver enzymes and cardiovascular disease risk; however, some of the liver enzyme-increasing variants associate with lower cardiovascular disease risk while others with higher risk. Some alleles that decrease liver enzymes also protect against cardiometabolic disease and thus medications causing a similar effect would be protective against both liver and heart diseases. For example, the ALT-increasing allele rs1277930-A (near *PSRC1*) associates with increased dyslipidemia and coronary artery disease at genome-wide significance for example. Another example is rs56094641-G (near *FTO*) is associated with increased diabetes, obesity, and dyslipidemia, and this variant was most significantly associated with BMI^35^. In contrast, the ALT-increasing allele rs58542926-T (*TM6SF2*) is associated with lower risk of dyslipidemia, the ALT-increasing rs429358-T (*APOE*) is associated with lower risk of ischemic heart disease and the AST- and ALP-increasing allele rs1260326-T (*GCKR*) associated with lower risk of diabetes. Thus targeting the genes may have protective effects on liver disease but may exacerbate disease in other organ systems.

Patterns of effects on metabolites may offer insights into disease pathophysiology. For example, specific alleles in variants in/near *APOE*, *LIPC*, *MLXIPL*, and *TM6SF2* promote liver disease while resulting in a favorable serum lipid profile of decreased TG/LDL or increased HDL, suggesting they may cause liver disease by sequestering lipids/metabolites in the liver. In contrast, liver enzyme-increasing *TRIB1* and *PSRC1* alleles are associated with an unfavorable serum lipid profile (higher TG/LDL or lower HDL), suggesting that targeting these genes may lead to improvement in liver function and lipid/lipoprotein metabolism. Our work also helps to identify patterns of metabolic effects that define the serum signature which can be used for better classification of disease subtypes in the future.

Recent advances in single cell RNA sequencing have made it possible to determine in which cell type a specific gene is expressed. Combining single cell sequencing and GWAS may suggest mechanisms by which a specific variant affects liver disease. For example, *TM6SF2* was expressed exclusively in hepatocytes, consistent with its reported effect on very low-density lipoprotein export in hepatocytes^36^. *PNPLA3*, in contrast, was expressed at high levels in both hepatocytes and cholangiocytes compared to other cell types. This finding is consistent with recent findings that *PNPLA3* interacts with Hedgehog-mediated cholangiocyte chemokine production^37,38^ in addition to its well-characterized effects on hepatocyte triglyceride export^39,40^. Some cell expression patterns are of genes that are well known to function in hepatocytes including ABC and SLC transporters, aldehyde dehydrogenases, *LEPR*, and *LIPC*. Other findings are previously-unreported, such as endothelial cell-specific expression of *PPARG* the function of which in this cell type remains to be determined. These findings may help generate hypotheses that can be the subject of further investigation. This is to our knowledge the first use of single cell RNA sequencing data in a GWAS of liver-related traits.

This study had several limitations. First, neither UKBB nor BBJ cohorts were population-based and thus our findings may not be generalizable to the general population in the UK and/or Japan. In addition, advanced liver disease was uncommon in both cohorts: cirrhosis prevalence was only 0.3% in UKBB^41^ and patients with chronic viral hepatitis, cirrhosis, or hepatocellular carcinoma were excluded from liver enzyme GWAS in BBJ^34^; such exclusions would reduce our power to identify liver disease. Second, only summary data from BBJ were available, not individual-level data. This limited our ability to control for certain covariates such as BMI or diabetes in that database. Also, we did not control for alcohol intake in UKBB. Reassuringly, only a small proportion of SNPs did not replicate in both studies, which suggests that despite these differences the disease biology identification across ancestries was robust. Finally, liver enzyme elevations can be seen in liver disease, metabolic disease such as hyperthyroidism, and with medication use. More in-depth phenotyping of participants may help identify these and further causes of elevated in liver tests.

Strengths of the study include that it assessed effects across ancestries which increases generalizability. It also characterized the effects of variants across human diseases, traits, tissues, genes and pathways providing further information for defining and treating human disease subtypes.

In conclusion, we have identified >300 previously-unreported genetic variants associated with circulating liver enzyme concentrations to date. Further analysis of these identified variants may contribute to our understanding of the genetics underlying liver disease, provide new targets for intervention, as well as lead to future identification of those most at risk for various liver diseases.

## Methods

### Ethics statement

All research in this study was approved by the Institutional Review Board of the University of Michigan (Ann Arbor, MI). UKBB protocols were approved by the National Research Ethics Service Committee and all participants provided written informed consent. Analyses in this project were conducted under UK BioBank Resource Project 18120. IRB approval was not required to use BBJ data as they are publicly-available. All MGI participants provided written informed consent approved by the University of Michigan Institutional Review Board (Ann Arbor, MI).

### Cohorts and genotyping

The UK BioBank includes genotypic, clinical, and demographic information of over 400,000 individuals aged 40–69 at time of recruitment. Genotyping and data collection has been previously described^42^. Participants were genotyped on one of two custom arrays: UK BiLEVE Axiom Array (*n* = 50,520) or UK BioBank Axiom Array (*n* = 438,692) with >95% overlap. We included only white-British participants. SNPs were imputed to the UK10K and Haplotype Reference Consortium. For quality control, we used EasyQC (version 9.2) with an imputation quality cutoff of 0.85. After quality control, 23,338,396 SNPs in >389,565 white-British individuals were included in genetic data for analysis.

The BioBank Japan cohort is as previously described^34^. SNPs were imputed to the Japanese population of the 1000 G Phase 1 panel^34^. We obtained publicly-available summary statistics from BioBank Japan per the Data Availability statement.

MGI is a hospital-based cohort of patients seen in Michigan Medicine (Ann Arbor, MI)^41,43^. ALT, AST, and ALP was determined based on the median value of all ambulatory ALT, AST, and ALP measurements for each individual participant (*n* = 35,730). Cirrhosis was defined based on imaging report of cirrhosis, liver biopsy showing stage 4 fibrosis, or diagnosis codes (ICD-9 571.5, 571.2, or 571.6, or ICD-10 K74.X, K70.2-4, or K71.7). Hepatic steatosis was defined based on liver biopsy or imaging showing steatosis. Participants were genotyped using an Illumina HumanCoreExome v.12.1 array, a GWAS and exome array with >500,000 SNPs. SNPs were imputed to Haplotype Reference Consortium release 1; imputed SNPS were excluded for poor imputation quality (*r*^2^ < 0.3) or minor allele count < 4. We included only Caucasian MGI participants as PRSs often transfer poorly across ancestries^44^.

### Genome-wide association study (GWAS) and meta-analysis

This study was a meta-analysis of UKBB and BBJ. In UKBB, we performed GWAS using a linear mixed model using SAIGE (version 0.29, https://github.com/weizhouUMICH/SAIGE) with inverse normally-transformed ALT, AST, or ALP as the dependent variable using an additive genetic model. Covariates were age, age^2^, sex, and principal components 1–10. BBJ GWAS results are as previously reported^15^; in brief, analyses were carried out using an additive genetic model with either log-transformed ALT or AST or rank-based inverse normalized ALP as the dependent variable. Models were adjusted for age, sex, principal components 1–10, alcohol intake, and status of 47 target diseases. Since we lacked individual-level data from BBJ, it was impossible to match the models in UKBB and BBJ, so we performed meta-analysis with UKBB and BBJ GWAS using METAL (28 Aug 2018 release, https://github.com/statgen/METAL) based on p values and sample size^16^. We excluded multi-allelic variants, indels, and variants with minor allele frequency <0.001 in the combined UKBB + BBJ population. We further calculated R^2^ between genome-wide significant (*p* < 5 × 10^−8^) SNPs within Japanese (1000 Genomes, Phase 1) or British populations (1000 Genomes plus UK10K).

Within each trait, SNPs within 1 Mb or *R*^2^ > 0.01 within either cohort of a lead SNP with lowest *p* value were removed to identify independent signals. Across traits, genome-wide significant SNPs were considered to represent the same locus if distance between them was <1 Mb. A SNP was considered novel if it was >1 Mb from SNPs previously reported to be associated at genome-wide significance with the same liver enzyme^12,13,45–48^.

Ancestry-specific variants were identified among variants that did not reach genome-wide significance in the meta-analysis but did within one of the two cohorts (i.e. UKBB or BBJ). Independent ancestry-specific variants were identified based on 1 MB distance from the lead variants in the METAL meta-analysis and from any other ancestry-specific variants for the specific trait. We excluded multi-allelic variants, indels, and variants with minor allele frequency < 0.001 in their respective populations.

Sex-specific variants were identified by separately conducting a GWAS in men and women for ALT, AST, and ALP in UKBB, and meta-analyzing the GWASes in METAL. We evaluated the alleles associated at genome-wide significance with ALT, AST, or ALP in the UKBB/BBJ meta-analysis to determine whether they demonstrated Bonferroni-adjusted significant heterogeneity (adjusted for 172 ALT, 199 AST, and 206 ALP alleles). We were not able to include BBJ in these analyses as individual-level data were not available.

### LD score regression

To estimate relative contributions of polygenicity and population stratification to genomic inflation, we conducted LD score regression using LDSC (version 1.0.0) as previously described^49^; LD score regression estimates heritability and genetic correlation from GWAS summary statistics.

### Directional consistency

To calculate directional consistency (DC) between UKBB and BBJ associations we assumed that the probability of *P(DC* | *associated)*=1 and *P(DC* | *not associated)*=0.5. In this formulation, *DC* = *R/2* + *S*, meaning that *S* = *2DC–T*, where *T* equals the total number of SNPs, *R* equals the number of SNPs not associated with the trait, and *S* equals the number of SNPs associated with the trait. With *DC* = 55 and *T* = 78, we estimate *S* to be 2*DC* – *T* = *2 ×* 55 – 78 = 32 for ALT, 2*DC* – *T* = *2 ×* 77 – 95 = 59 for AST, and 2*DC* – *T* = *2 ×* 96 – 138 = 54 for ALP.

### Data-driven Expression Prioritization Integration for Complex Traits (DEPICT)

DEPICT prioritizes tissues, genes, and pathways highlighted by GWAS analyses^21^. All independent variants associated with ALT, AST, or ALP on meta-analysis (*p* < 5 × 10^−8^) were used as input for DEPICT (version 3, 29 June 2007 release, https://data.broadinstitute.org/mpg/depict/). Tissue or gene set enrichment was considered statistically significant at a false discovery rate *p*-value <0.05. Cytoscape (version 3.7.1) was used for creating plots and visualization for UK BioBank complex networks from DEPICT output. It is available at https://cytoscape.org/.

### Phenome-wide association study (PheWAS)

PheWAS was performed for genetic variants associated with ALT, AST, or ALP at genome-wide significance in the meta-analysis using previously-published data^22^. Only diseases with an associated International Classification of Diseases-10 diagnosis code were included in PheWAS. In situations where a variant was associated with both a more specific and a less specific diagnosis code, only the more specific code was used. For example, if a variant was associated with both “N20-N23 Urolithiasis” and “N20 Calculus of kidney and ureter”, only the latter was reported. A cutoff of *p* < 5 × 10^−8^ for variant-disease associations was used for statistical significance.

### Latent causal variable

Latent causal variable is a method that estimates direction of causality between traits^24^. In brief, this model assumes that a latent causal variable mediates the genetic correlation between two traits. If trait 1 is causal for trait 2, then alleles with large effects on trait 1 (large $\alpha _1^2$) will also have large effects on trait 2 (large *α*_1_*α*_2_), but alleles with large effects on trait 2 (large $\alpha _2^2$) will not have correspondingly large effects on trait 1 (small *α*_1_*α*_2_). We excluded all SNPs with minor allele frequency <0.05 and that were not present in GWAS for both the liver enzyme and the metabolic trait^24^. Only traits with high heritability (Z score for heritability > 7) were used in subsequent analysis^24^.

### Liver disease traits

We obtained summary statistics from previously-published GWAS for liver attenuation (higher liver attenuation implies less hepatic steatosis)^25^, alcoholic cirrhosis^26^, primary biliary cholangitis^27^, and primary sclerosing cholangitis^28^.

### Polygenic risk scores

We created PRSs for ALT, AST, and ALP using only the alleles associated with the respective liver enzymes at genome-wide significance (Supplementary Data 1–3). These PRSs were weighted by the beta value of each allele on ALT, AST, or ALP in UKBB. We identified SNPs present in both UKBB and MGI, excluded all potentially-ambiguous SNPs (A/T or C/G), and selected independent SNPs based on 1 MB distance and *R*^2^ < 0.01 as earlier. The ALT PRS consisted of 172 independent alleles, the AST PRS of 198, and the ALP PRS of 215. We then examined associations between the three PRSs and rank-based inverse-normally transformed liver enzyme concentrations (continuous variable and linear regression), and cirrhosis and steatosis in MGI (binary variable and logistic regression). PRSs were defined either as inverse-normally transformed rank units or as percentiles. All analyses were adjusted for age, age^2^, sex, and principal components 1–10.

### Mendelian diseases

We identified all non-synonymous variants within LD (*R*^2^ > 0.8) of liver enzyme-increasing variants using the UK10K reference panel. We then searched the OMIM Clinical Synopsis section for the genes that these non-synonymous variants were in, including only traits with documented inheritance and molecular basis. When the variants that have previously been reported to be associated with Mendelian disease were available in UKBB, we determined whether the effect of the non-synonymous LD variants were independent of these Mendelian variants by performing regression in UKBB with the inverse normally-transformed liver enzyme adjusted for the (available) previously-reported Mendelian variants, sex, age, age^2^, and principal components 1–10.

### Single cell RNA sequencing analysis

We assigned to each variant the nearest gene and assessed cell expression of each variant based on previously-published single cell RNA sequencing data and annotations (GEO accession GSE124395)^29^. We included five populations of cells: hepatocytes, cholangiocytes, Kupffer cells, endothelial cells (including liver sinusoidal endothelial cells, microvascular endothelial cells, and other endothelial cells), and natural killer/T/natural killer-T cells. Differential gene analysis was performed using Wilcoxon rank-sum tests. Genes were considered more highly expressed in a cell type if the mean expression in that cell type was at least double the mean of all other cell types and statistically significantly-different from that of all other cell types. Statistical significance was based on Bonferroni correction for 33,941 (total number of genes available^29^) times 10 (five two-way comparisons between the five cell types listed above) comparisons, i.e. *p* < 1.5 × 10^−7^.

### Reporting summary

Further information on research design is available in the Nature Research Reporting Summary linked to this article.

## Supplementary information

Supplementary Information

Description of Additional Supplementary Files

Supplementary Data 1

Supplementary Data 2

Supplementary Data 3

Supplementary Data 4

Supplementary Data 5

Supplementary Data 6

Supplementary Data 7

Supplementary Data 8

Supplementary Data 9

Supplementary Data 10

Supplementary Data 11

Supplementary Data 12

Supplementary Data 13

Supplementary Data 14

Supplementary Data 15

Supplementary Data 16

Supplementary Data 17

Supplementary Data 18

Supplementary Data 19

Supplementary Data 20

Supplementary Data 21

Supplementary Data 22

Supplementary Data 23

Supplementary Data 24

Supplementary Data 25

Supplementary Data 26

Supplementary Data 27

Supplementary Data 28

Supplementary Data 29

Supplementary Data 30

Supplementary Data 31

Reporting Summary

## Data Availability

Meta-analysis results from this study are available at GWAS Catalog: accession GCST90011898 for ALT (ftp://ftp.ebi.ac.uk/pub/databases/gwas/summary_statistics/GCST90011898/), GCST90011899 for AST (ftp://ftp.ebi.ac.uk/pub/databases/gwas/summary_statistics/GCST90011899/), and GCST90011900 for ALP (ftp://ftp.ebi.ac.uk/pub/databases/gwas/summary_statistics/GCST90011900/). They are also available at http://www.med.umich.edu/spelioteslab/. UK BioBank genomic and phenotypic data supporting this publication are available upon application (https://ukbiobank.ac.uk). BioBank Japan data are available at the National Bioscience Database Center Human Database (hum0014, https://humandbs.biosciencedbc.jp/en/hum0014-v6). Summary statistics on liver attenuation are from^23^, alcoholic cirrhosis from^26^ (http://gengastro.med.tu-dresden.de/suppl/alc_cirrhosis/), primary biliary cholangitis from^24^ (Supplementary Table 17), and primary sclerosing cholangitis from^25^ (https://www.ipscsg.org/published-studies/). Michigan Genomics Initiative individual-level data are not currently available to the public due to patient privacy requirements. Otherwise, all data used to generate figures can be found in supplementary tables.
