# Author Correction: Genome-wide association study of serum liver enzymes implicates diverse metabolic and liver pathology

**DOI:** 10.1038/s41467-023-38659-3

**Published:** 2023-06-08

**Authors:** Vincent L. Chen, Xiaomeng Du, Yanhua Chen, Annapurna Kuppa, Samuel K. Handelman, Rishel B. Vohnoutka, Patricia A. Peyser, Nicholette D. Palmer, Lawrence F. Bielak, Brian Halligan, Elizabeth K. Speliotes

**Affiliations:** 1 Division of Gastroenterology and Hepatology, University of Michigan Health System, Ann Arbor, MI USA; 2 Department of Computational Medicine and Bioinformatics, University of Michigan Medical School, Ann Arbor, MI USA; 3 Department of Epidemiology, University of Michigan School of Public Health, Ann Arbor, MI USA; 4 Department of Biochemistry, Wake Forest School of Medicine, Winston-Salem, NC USA

**Keywords:** Genome-wide association studies, Biomarkers, Liver diseases

Correction to: *Nature Communications* 10.1038/s41467-020-20870-1, published online 05 February 2021

The original version of this Article contained multiple errors in Figure 5. Genes “TMEM263, TRIM59” were incorrectly categorized in red boxes, while genes “CDKL1A, GRB1, MCM6, UBD, VIM-AS1, C5orf67, FRK, PPARD” were inadvertently omitted from the original figure. These mistakes were due to underlying errors in Supplementary Table 17 and Supplementary Data 20–22.

The correct version of Fig. 5 is:
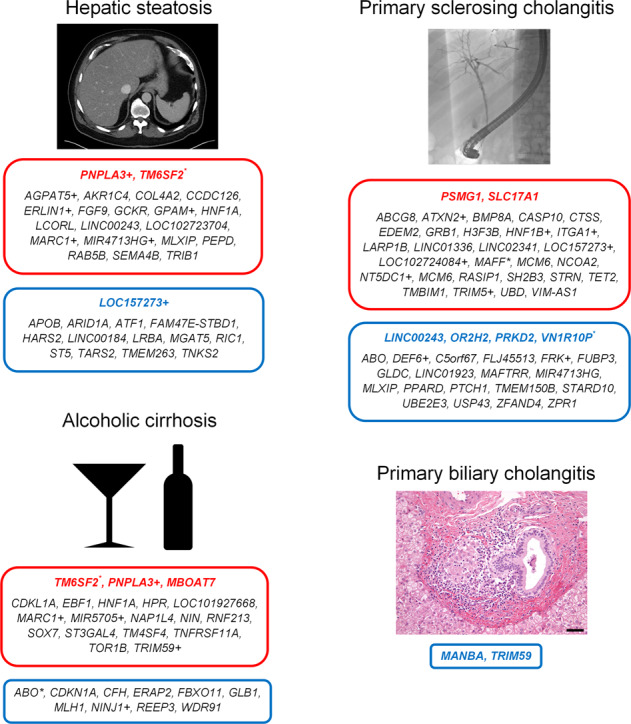


which replaces the previous incorrect version:
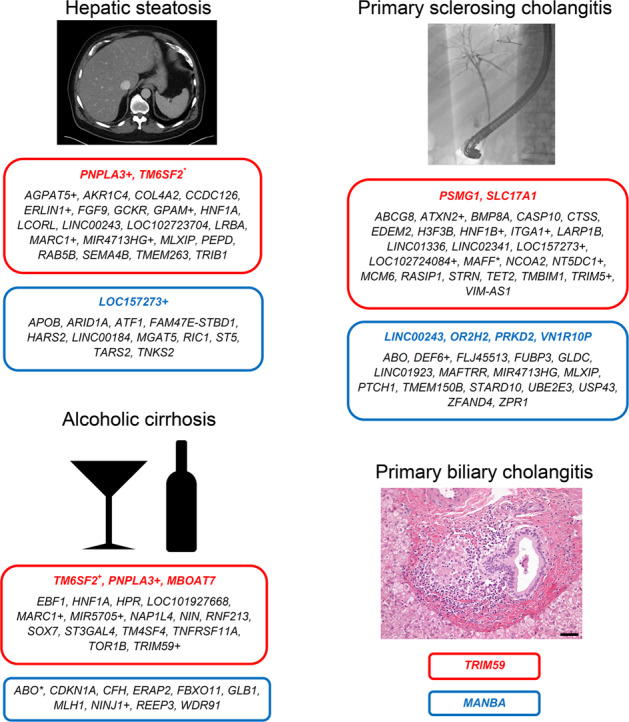


Supplementary Table 17 and Supplementary Data 20–22 have been updated to correct these errors. The original incorrect version of Supplementary Table 17 and Supplementary Data 20–22 can be found as Supplementary information associated with this Correction. These errors have been corrected in the HTML and PDF versions of the Article.

## Supplementary information

Updated Supplementary Data 20

Updated Supplementary Data 21

Updated Supplementary Data 22

Updated Supplementary Information

Incorrect Supplementary Data 20

Incorrect Supplementary Data 21

Incorrect Supplementary Data 22

Incorrect Supplementary Information

