# Supplementary material for: Genome-wide association study of serum liver enzymes implicates diverse metabolic and liver pathology
Source: Nat Commun. 2021 Feb 5;12:816. doi: 10.1038/s41467-020-20870-1 (PMC7865025; doi:10.1038/s41467-020-20870-1)
Supplement: Supplementary file 2 — Description of Additional Supplementary Files [file 41467_2020_20870_MOESM2_ESM.pdf]

Description of Additional Supplementary Files

File Name: Supplementary Data 1.

Description: CHR:POS, chromosome:position. EA, effect allele. OA, other allele. EAF, effect allele frequency. Gene tags: (e) exonic, (i) intronic, (u) upstream, (d) downstream, (inter) intergenic, (UTR) untranslated region.

File Name: Supplementary Data 2.

Description: CHR:POS, chromosome:position. EA, effect allele. OA, other allele. EAF, effect allele frequency. Gene tags: (e) exonic, (i) intronic, (u) upstream, (d) downstream, (inter) intergenic, (UTR) untranslated region.

File Name: Supplementary Data 3.

Description: CHR:POS, chromosome:position. EA, effect allele. OA, other allele. EAF, effect allele frequency. Gene tags: (e) exonic, (i) intronic, (u) upstream, (d) downstream, (inter) intergenic, (UTR) untranslated region.

File Name: Supplementary Data 4.

Description: CHR:POS, chromosome:position. EA, effect allele. OA, other allele. EAF, effect allele frequency. ALT, alanine aminotransferase. AST, aspartate aminotransferase. ALP, alkaline phosphatase. Gene tags: (e) exonic, (i) intronic, (u) upstream, (d) downstream, (inter) intergenic, (UTR) untranslated region.

File Name: Supplementary Data 5.

Description: CHR:POS, chromosome:position. EA, effect allele. OA, other allele. EAF, effect allele frequency. Gene tags: (e) exonic, (i) intronic, (u) upstream, (d) downstream, (inter) intergenic, (UTR) untranslated region.

File Name: Supplementary Data 6.

Description: CHR:POS, chromosome:position. EA, effect allele. OA, other allele. EAF, effect allele frequency. Gene tags: (e) exonic, (i) intronic, (u) upstream, (d) downstream, (inter) intergenic, (UTR) untranslated region.

File Name: Supplementary Data 7.

Description: CHR:POS, chromosome:position. EA, effect allele. OA, other allele. EAF, effect allele frequency. Gene tags: (e) exonic, (i) intronic, (u) upstream, (d) downstream, (inter) intergenic, (UTR) untranslated region.

File Name: Supplementary Data 8.

Description: DEPICT, Data-driven Expression-Prioritized Integration for Complex Traits. MeSH, Medical Subject Headings.

File Name: Supplementary Data 9.

Description: DEPICT, Data-driven Expression-Prioritized Integration for Complex Traits. MeSH, Medical Subject Headings.

File Name: Supplementary Data 10.

Description: DEPICT, Data-driven Expression-Prioritized Integration for Complex Traits. MeSH, Medical Subject Headings.

File Name: Supplementary Data 11.

Description: Only gene sets enriched at false discovery rate < 0.05 are shown. DEPICT, Data-driven Expression-Prioritized Integration for Complex Traits. ENSG, Ensembl Gene. GO, Gene Ontology. KEGG, Kyoto Encylopedia of Genes and Genomes. MP, Mouse Phenotype.

File Name: Supplementary Data 12.

Description: Only gene sets enriched at false discovery rate < 0.05 are shown. DEPICT, Data-driven Expression-Prioritized Integration for Complex Traits. ENSG, Ensembl Gene. GO, Gene Ontology. KEGG, Kyoto Encylopedia of Genes and Genomes. MP, Mouse Phenotype.

File Name: Supplementary Data 13.

Description: Only gene sets enriched at false discovery rate < 0.05 are shown. DEPICT, Data-driven Expression-Prioritized Integration for Complex Traits. ENSG, Ensembl Gene. GO, Gene Ontology. KEGG, Kyoto Encylopedia of Genes and Genomes. MP, Mouse Phenotype.

File Name: Supplementary Data 14.

Description: CHR:POS, chromosome:position. EA, effect allele. OA, other allele. EAF, effect allele frequency. Gene tags: (e) exonic, (i) intronic, (u) upstream, (d) downstream, (inter) intergenic, (UTR) untranslated region. Trait labels: BMI, body mass index; DBP, diastolic blood pressure; HDL, high-density lipoproteins; LDL, low-density lipoproteins; SBP, systolic blood pressure; TG, triglycerides, WC, waist circumference; WHRADJBMI, waist-to-hip ratio adjusted for body mass index; WHR, waist-to-hip ratio.

File Name: Supplementary Data 15.

Description: CHR:POS, chromosome:position. EA, effect allele. OA, other allele. EAF, effect allele frequency. Gene tags: (e) exonic, (i) intronic, (u) upstream, (d) downstream, (inter) intergenic, (UTR) untranslated region. Trait labels: BMI, body mass index; DBP, diastolic blood pressure; HDL, high-density lipoproteins; LDL, low-density lipoproteins; SBP, systolic blood pressure; TG, triglycerides, WC, waist circumference; WHRADJBMI, waist-to-hip ratio adjusted for body mass index; WHR, waist-to-hip ratio.

File Name: Supplementary Data 16.

Description: CHR:POS, chromosome:position. EA, effect allele. OA, other allele. EAF, effect allele frequency. Gene tags: (e) exonic, (i) intronic, (u) upstream, (d) downstream, (inter) intergenic, (UTR) untranslated region. Trait labels: BMI, body mass index; DBP, diastolic blood pressure; HDL, high-density lipoproteins; LDL, low-density lipoproteins; SBP, systolic blood pressure; TG, triglycerides, WC, waist circumference; WHRADJBMI, waist-to-hip ratio adjusted for body mass index; WHR, waist-to-hip ratio.

File Names: Supplementary Data 17-19.

Description: Z scores of associations between metabolites and liver enzyme-increasing variants. Missing values are left blank. AcAce, Acetoacetate Ace, Acetate. Ala, Alanine. Alb, Albumin. Bis.DB.ratio, Ratio of bis-allylic bonds to double bonds in lipids. Bis.FA.ratio, Ratio of bis-allylic bonds to total fatty acids in lipids. bOHBut, 3Lhydroxybutyrate. CH2.DB.ratio, CH2 groups in fatty acids. CH2.in.FA, CH2 groups to double bonds ratio. Cit, Citrate. Crea, Creatinine. DB.in.FA, Double bonds in fatty acids. DHA, 22:6 docosahexaenoic acid (DHA). Est.C, Esterified cholesterol. FALen, Fatty acid length. FAw3, Omega-3 fatty acids. FAw6, Omega-6 fatty acids. FAw79S, Omega-7 and -9 and saturated fatty acids. Free.C, Free cholesterol. Glc, Glucose. Gln, Glutamine. Glol, Glycerol. Gly, Glycine. Gp, Glycoprotein acetyls mainly a1-acid glycoprotein. HDL.C, Total cholesterol in high-density lipoproteins. HDL.D, high-density lipoprotein diameter. His, Histidine. IDL.C, Total cholesterol in intermediate-density lipoproteins. IDL.FC, Free cholesterol in intermediate-density lipoproteins. IDL.L, Total lipids in intermediate-density lipoproteins. IDL.P, Concentration of intermediate-density lipoprotein particles. IDL.PL, Phospholipids in intermediate-density lipoproteins. IDL.TG, Triglycerides in intermediate-density lipoproteins. Ile, Isoleucine. L.HDL.C, Total cholesterol in large high-density lipoproteins. L.HDL.CE, Cholesterol esters in large high-density lipoproteins. L.HDL.FC, Free cholesterol in large high-density lipoproteins. L.HDL.L, Total lipids in large high-density lipoproteins. L.HDL.P, Concentration of large high-density lipoprotein particles. L.HDL.PL, Phospholipids in large high-density lipoproteins. L.LDL.C, Total cholesterol in large low-density lipoproteins. L.LDL.CE, Cholesterol esters in large low-density lipoproteins. L.LDL.FC, Free cholesterol in large low-density lipoproteins. L.LDL.L, Total lipids in large low-density lipoproteins. L.LDL.P, Concentration of large low-density lipoprotein particles.

L.LDL.PL, Phospholipids in large low-density lipoproteins. L.VLDL.C, Total cholesterol in large very low-density lipoproteins. L.VLDL.CE, Cholesterol esters in large very low-density lipoproteins. L.VLDL.FC, Free cholesterol in large very low-density lipoproteins. L.VLDL.L, Total lipids in large very low-density lipoproteins. L.VLDL.P, Concentration of large very low-density lipoprotein particles. L.VLDL.PL, Phospholipids in large very low-density lipoproteins. L.VLDL.TG, Triglycerides in large very low-density lipoproteins. LA, 18:2 linoleic acid. Lac, Lactate. LDL.C, Total cholesterol in low-density

lipoproteins. LDL.D, low-density lipoprotein diameter. Leu, Leucine. M.HDL.C, Total cholesterol in medium high-density lipoproteins. M.HDL.CE, Cholesterol esters in medium high-density lipoproteins. M.HDL.FC, Free cholesterol in medium high-density lipoproteins. M.HDL.L, Total lipids in medium high-density lipoproteins. M.HDL.P, Concentration of medium high-density lipoprotein particles. M.HDL.PL, Phospholipids in medium high-density lipoproteins. M.LDL.C, Total cholesterol in medium low-density lipoproteins. M.LDL.CE, Cholesterol esters in medium low-density lipoproteins. M.LDL.L, Total lipids in medium low-density lipoproteins. M.LDL.P, Concentration of medium low-density lipoprotein particles. M.LDL.PL, Phospholipids in medium low-density lipoproteins. M.VLDL.C, Total cholesterol in medium very low-density lipoproteins. M.VLDL.CE, Cholesterol esters in medium very low-density lipoproteins. M.VLDL.FC, Free cholesterol in medium very low-density lipoproteins. M.VLDL.L, Total lipids in medium very low-density lipoproteins. M.VLDL.P, Concentration of medium very low-density lipoprotein particles. M.VLDL.PL, Phospholipids in medium very low-density lipoproteins. M.VLDL.TG, Triglycerides in medium very low-density lipoproteins. MUFA, Mono-unsaturated fatty acids. otPUFA, Other polyunsaturated fatty acids than 18:2. PC, Phosphatidylcholine and other cholines. Phe, Phenylalanine. Pyr, Pyruvate. S.HDL.L, Total lipids in small high-density lipoproteins. S.HDL.P, Concentration of small high-density lipoprotein particles. S.HDL.TG, Triglycerides in small high-density lipoproteins. S.LDL.C, Total cholesterol in small low-density lipoproteins. S.LDL.L, Total lipids in small low-density lipoproteins. S.LDL.P, Concentration of small low-density lipoprotein particles. S.VLDL.C, Total cholesterol in small very low-density lipoproteins. S.VLDL.FC, Free cholesterol in small very low-density lipoproteins. S.VLDL.L, Total lipids in small very low-density lipoproteins. S.VLDL.P, Concentration of small very low-density lipoprotein particles. S.VLDL.PL, Phospholipids in small very low-density lipoproteins. S.VLDL.TG, Triglycerides in small very low-density lipoproteins. Serum.C, Serum total cholesterol. Serum.TG, Serum total triglycerides. SM, Sphingomyelins. Tot.FA, Total fatty acids. TotPG, Total phosphoglycerides. Tyr, Tyrosine. Urea, Urea. Val, Valine. VLDL.D, very low-density lipoprotein diameter.

XL.HDL.C, Total cholesterol in very large high-density lipoproteins. XL.HDL.CE, Cholesterol esters in very large high-density lipoproteins. XL.HDL.FC, Free cholesterol in very large high-density lipoproteins. XL.HDL.L, Total lipids in very large high-density lipoproteins. XL.HDL.P, Concentration of very large high-density lipoprotein particles. XL.HDL.PL, Phospholipids in very large high-density lipoproteins. XL.HDL.TG, Triglycerides in very large high-density lipoproteins. XL.VLDL.L, Total lipids in very large very low-density lipoproteins. XL.VLDL.P, Concentration of very large very low-density lipoprotein particles. XL.VLDL.PL, Phospholipids in very large very low-density lipoproteins. XL.VLDL.TG, Triglycerides in very large very low-density lipoproteins. XS.VLDL.L, Total lipids in very small very low-density lipoproteins. XS.VLDL.P, Concentration of very small very low-density lipoprotein particles. XS.VLDL.PL, Phospholipids in very small very low-density lipoproteins. XS.VLDL.TG, Triglycerides in very small very low-density lipoproteins. XXL.VLDL.L, Total lipids in chylomicrons and extremely large very low-density lipoproteins. XXL.VLDL.P, Concentration of chylomicrons and extremely large very low-density lipoprotein particles. XXL.VLDL.PL, Phospholipids in chylomicrons and extremely large very low-density lipoproteins. XXL.VLDL.TG, Triglycerides in chylomicrons and extremely large very low-density lipoproteins.

File Name: Supplementary Data 20.

Description: CHR:POS, chromosome:position. EA, effect allele. OA, other allele. EAF, effect allele frequency. When liver enzyme-increasing variants were not themselves available, proxy variants

were used. EA and OA of proxy variants are shown in parentheses. Gene tags: (e) exonic, (i) intronic, (u) upstream, (d) downstream, (inter) intergenic, (UTR) untranslated region.

File Name: Supplementary Data 21.

Description: CHR:POS, chromosome:position. EA, effect allele. OA, other allele. EAF, effect allele frequency. When liver enzyme-increasing variants were not themselves available, proxy variants were used. EA and OA of proxy variants are shown in parentheses. Gene tags: (e) exonic, (i) intronic, (u) upstream, (d) downstream, (inter) intergenic, (UTR) untranslated region.

File Name: Supplementary Data 22.

Description: CHR:POS, chromosome:position. EA, effect allele. OA, other allele. EAF, effect allele frequency. When liver enzyme-increasing variants were not themselves available, proxy variants were used. EA and OA of proxy variants are shown in parentheses. Gene tags: (e) exonic, (i) intronic, (u) upstream, (d) downstream, (inter) intergenic, (UTR) untranslated region.

File Name: Supplementary Data 23.

Description: CHR:POS, chromosome:position. EA, effect allele. OA, other allele. EAF, effect allele frequency. When liver enzyme-increasing variants were not themselves available, proxy variants were used. EA and OA of proxy variants are shown in parentheses. Gene tags: (e) exonic, (i) intronic, (u) upstream, (d) downstream, (inter) intergenic, (UTR) untranslated region.

File Name: Supplementary Data 24.

Description: CHR:POS, chromosome:position. EA, effect allele. OA, other allele. EAF, effect allele frequency. When liver enzyme-increasing variants were not themselves available, proxy variants were used. EA and OA of proxy variants are shown in parentheses. Gene tags: (e) exonic, (i) intronic, (u) upstream, (d) downstream, (inter) intergenic, (UTR) untranslated region.

File Name: Supplementary Data 25.

Description: CHR:POS, chromosome:position. EA, effect allele. OA, other allele. EAF, effect allele frequency. When liver enzyme-increasing variants were not themselves available, proxy variants were used. EA and OA of proxy variants are shown in parentheses. Gene tags: (e) exonic, (i) intronic, (u) upstream, (d) downstream, (inter) intergenic, (UTR) untranslated region.

File Name: Supplementary Data 26.

Description: CHR:POS, chromosome:position. EA, effect allele. OA, other allele. EAF, effect allele frequency. When liver enzyme-increasing variants were not themselves available, proxy variants were used. EA and OA of proxy variants are shown in parentheses. Gene tags: (e) exonic, (i) intronic, (u) upstream, (d) downstream, (inter) intergenic, (UTR) untranslated region.

File Name: Supplementary Data 27.

Description: CHR:POS, chromosome:position. EA, effect allele. OA, other allele. EAF, effect allele frequency. When liver enzyme-increasing variants were not themselves available, proxy variants were used. EA and OA of proxy variants are shown in parentheses. Gene tags: (e) exonic, (i) intronic, (u) upstream, (d) downstream, (inter) intergenic, (UTR) untranslated region.

File Name: Supplementary Data 28.

Description: CHR:POS, chromosome:position. EA, effect allele. OA, other allele. EAF, effect allele frequency. When liver enzyme-increasing variants were not themselves available, proxy variants were used. EA and OA of proxy variants are shown in parentheses. Gene tags: (e) exonic, (i) intronic, (u) upstream, (d) downstream, (inter) intergenic, (UTR) untranslated region.

File Name: Supplementary Data 29.

Description: LD, linkage disequilibrium. CHR:POS, chromosome:position. "Covariates" column indicates Mendelian disease variants that were present in UK BioBank. Bold denotes that the liver enzyme-affecting variant influences liver enzymes independently of previously-reported Mendelian disease-causing variants. Italics denotes that the liver enzyme-affecting variant is the same as a previously-reported Mendelian disease-causing variant.

File Name: Supplementary Data 30.

Description: Mean expression of genes and coding genes nearest to liver enzyme-increasing variants, based on cell type. NK, natural killer. Significance: q, significantly different in hepatocytes vs. cholangiocytes; r, hepatocytes vs. endothelial cells; s, hepatocytes vs. Kupffer cells; t, hepatocytes vs. NK/T/NKT cells; u, cholangiocytes vs. endothelial cells; v, cholangiocytes vs. Kupffer cells; w, cholangiocytes vs. NK/T/NKT cells; x, endothelial cells vs. Kupffer cells; y, endothelial cells vs. NK/T/NKT cells; z, Kupffer cells vs. NK/T/NKT cells. Specificity: h, two-fold higher expression in hepatocytes vs. other cell types; c, in cholangiocytes; e, in endothelial cells; k, in Kupffer cells; n, in NK/T/NKT cells.

File Name: Supplementary Data 31.

Description: nkt, natural killer, natural killer-T, or T cell. lsec, liver sinusoidal endothelial cell. mvec, microvascular endothelial cell. endoth, other endothelial cell.
