# Supplementary material for: Genome-wide association study of serum liver enzymes implicates diverse metabolic and liver pathology
Source: Nat Commun. 2021 Feb 5;12:816. doi: 10.1038/s41467-020-20870-1 (PMC7865025; doi:10.1038/s41467-020-20870-1)
Supplement: Supplementary file 34 — Reporting Summary [file 41467_2020_20870_MOESM34_ESM.pdf]

nature research

Corresponding author(s): Elizabeth K Speliotes

Last updated by author(s): 1/17/2021

Reporting Summary

Nature Research wishes to improve the reproducibility of the work that we publish. This form provides structure for consistency and transparency in reporting. For further information on Nature Research policies, see our Editorial Policies and the Editorial Policy Checklist.

Statistics

For all statistical analyses, confirm that the following items are present in the figure legend, table legend, main text, or Methods section.

n/a	Confirmed	
☐	☒	The exact sample size (*n*) for each experimental group/condition, given as a discrete number and unit of measurement
☐	☒	A statement on whether measurements were taken from distinct samples or whether the same sample was measured repeatedly
☐	☒	The statistical test(s) used AND whether they are one- or two-sided<br>*Only common tests should be described solely by name; describe more complex techniques in the Methods section.*
☐	☒	A description of all covariates tested
☐	☒	A description of any assumptions or corrections, such as tests of normality and adjustment for multiple comparisons
☐	☒	A full description of the statistical parameters including central tendency (e.g. means) or other basic estimates (e.g. regression coefficient) AND variation (e.g. standard deviation) or associated estimates of uncertainty (e.g. confidence intervals)
☐	☒	For null hypothesis testing, the test statistic (e.g. *F*, *t*, *r*) with confidence intervals, effect sizes, degrees of freedom and *P* value noted<br>*Give P values as exact values whenever suitable.*
☒	☐	For Bayesian analysis, information on the choice of priors and Markov chain Monte Carlo settings
☒	☐	For hierarchical and complex designs, identification of the appropriate level for tests and full reporting of outcomes
☒	☐	Estimates of effect sizes (e.g. Cohen's *d*, Pearson's *r*), indicating how they were calculated

Our web collection on statistics for biologists contains articles on many of the points above.

Software and code

Policy information about availability of computer code

Data collection	For quality control of UK BioBank genotypic data, we used EasyQC 9.2 (http://www.genepi-regensburg.de/easyqc). Data from BioBank Japan were collected and analyzed as previously reported (PMID 29403010) and we used the summary statistics provided available at the National Bioscience Database Center Human Database (hum0014). Data management was performed in R, version 3.5.1. All software programs used are available for public use and no custom code was employed.
Data analysis	SAIGE were used to perform single variant association tests of UK BioBank data, and is available at https://github.com/weizhouUMICH/SAIGE. METAL (28 Aug 2018 release) was used for meta-analysis of UK BioBank and BioBank Japan summary data, and is available at http://csg.sph.umich.edu/abecasis/METAL. LDSC (version 1.0.0) was used for estimating heritability (Lambda GC and intercepts), genetic correlation, and LD score regression intercept based on UK BioBank and BioBank Japan summary data, and is available at https://github.com/bulik/ldsc. DEPICT (version 3, 29 June 2007 release) was used for gene prioritization and tissue/pathway enrichment analysis and is available for download at https://data.broadinstitute.org/mpg/depict. Cytoscape (version 3.7.1) was used to create plots for visualization of DEPICT output; it is available as https://cytoscape.org. Latent Causal Variable model was used to estimate direction of causality between traits, and is available at https://github.com/lukejoconnor/LCV. Other analyses were performed using R, version 3.5.1 and Python 2.7.

For manuscripts utilizing custom algorithms or software that are central to the research but not yet described in published literature, software must be made available to editors and reviewers. We strongly encourage code deposition in a community repository (e.g. GitHub). See the Nature Research guidelines for submitting code & software for further information.

Data

Policy information about availability of data

All manuscripts must include a data availability statement. This statement should provide the following information, where applicable:

- Accession codes, unique identifiers, or web links for publicly available datasets
- A list of figures that have associated raw data
- A description of any restrictions on data availability

Meta-analysis results from this study are available at GWAS Catalog: accession GCST90011898 for ALT (ftp://ftp.ebi.ac.uk/pub/databases/gwas/summary_statistics/GCST90011898/), GCST90011899 for AST (ftp://ftp.ebi.ac.uk/pub/databases/gwas/summary_statistics/GCST90011899/), and GCST90011900 for ALP (ftp://ftp.ebi.ac.uk/pub/databases/gwas/summary_statistics/GCST90011900/). They are also available at http://www.med.umich.edu/spelioteslab/. UK BioBank genomic and phenotypic data supporting this publication are available upon application (https://ukbiobank.ac.uk). BioBank Japan data are available at the National Bioscience Database Center Human Database (hum0014, https://humandbs.biosciencedbc.jp/en/hum0014-v6). Summary statistics on liver attenuation are from 23, alcoholic cirrhosis from 24 (http://gengastro.med.tu-dresden.de/suppl/alc_cirrhosis/), primary biliary cholangitis from 25 (Supplementary Table 17), and primary sclerosing cholangitis from 26 (https://www.ipscsg.org/published-studies/). Michigan Genomics Initiative individual-level data are not currently available to the public due to patient privacy requirements. Otherwise, all data used to generate figures can be found in supplementary tables.

Field-specific reporting

Please select the one below that is the best fit for your research. If you are not sure, read the appropriate sections before making your selection.

[x] Life sciences [ ] Behavioural & social sciences [ ] Ecological, evolutionary & environmental sciences

For a reference copy of the document with all sections, see nature.com/documents/nr-reporting-summary-flat.pdf

Life sciences study design

All studies must disclose on these points even when the disclosure is negative.

Sample size	Sample sizes were determined by the number of enrolled participants in UK BioBank (N = 389,565-390,812 for primary analyses) and BioBank Japan (N = 162,255). These were among the largest publicly-available datasets.
Data exclusions	Participants: Only Caucasian individuals from UK BioBank were included to facilitate genetic analyses and because an independent non-Caucasian population was available via BioBank Japan. All BioBank Japan participants were included.<br><br>Genotypes: In UK BioBank, all single nucleotide polymorphisms with imputation quality > 0.85 were included; triallelic variants, insertion-deletions, and very rare variants (minor allele frequency < 0.001) were excluded. Details on exclusions in BioBank Japan can be found in PMID 29403010. In the meta-analysis, triallelic variants, insertion-deletions, and very rare variants (minor allele frequency < 0.001) were excluded.<br><br>These exclusions were pre-specified.
Replication	We used two cohorts of different ethnic backgrounds and used meta-analysis to ensure robust and reproducible identification of genetic variants associated with liver enzyme concentrations. Attempts at replication were successful (see Supp. Data 1-3 which provide the list of variants used in the rest of the manuscript) except where explicitly specified otherwise (Supp. Data 5-7, Supp. Tables 6-7).
Randomization	This was an observational cohort study and therefore randomization was not required.
Blinding	This was an observational cohort study and therefore blinding was not required.

Reporting for specific materials, systems and methods

We require information from authors about some types of materials, experimental systems and methods used in many studies. Here, indicate whether each material, system or method listed is relevant to your study. If you are not sure if a list item applies to your research, read the appropriate section before selecting a response.

Materials & experimental systems

n/a	Involved in the study	
[x]	[ ]	Antibodies
[x]	[ ]	Eukaryotic cell lines
[x]	[ ]	Palaeontology and archaeology
[x]	[ ]	Animals and other organisms
[ ]	[x]	Human research participants
[x]	[ ]	Clinical data
[x]	[ ]	Dual use research of concern

Methods

n/a	Involved in the study	
[x]	[ ]	ChIP-seq
[x]	[ ]	Flow cytometry
[x]	[ ]	MRI-based neuroimaging

Human research participants

Policy information about studies involving human research participants

Population characteristics	In the UK BioBank cohort, median age was 67 years, 54% of participants were female, and all participants were Caucasian. In BioBank Japan, mean age was 62.7 years for men and 61.5 years for women; 53% were male; and all participants were of East Asian ancestry. In the Michigan Genomics Initiative cohort, median age was 59 years, 53% of participants were female, and all participants were Caucasian.
Recruitment	Participants were recruited to UK BioBank via mail sent to United Kingdom residents aged 40-69 years, and then enrolled at one of 22 centers. BioBank Japan participants were recruited between June 2003 and March 2008 among adults seen at one of 66 hospitals in Japan. Michigan Genomics Initiative participants were recruited among patients undergoing elective surgery at Michigan Medicine (Ann Arbor, MI, USA). These may result in selection biases such that UK BioBank participants (recruited from the general population) are likely to be healthier on average than BioBank Japan and Michigan Genomics Initiative participants (recruited from hospitals).
Ethics oversight	All research in this study was approved by the Institutional Review Board of the University of Michigan (Ann Arbor, MI). UKBB protocols were approved by the National Research Ethics Service Committee and all participants provided written informed consent. Analyses in this project were conducted under UK BioBank Resource Project 18120. IRB approval was not required to use BBJ data as they are publicly-available. All MGI participants provided written informed consent approved by the University of Michigan Institutional Review Board (Ann Arbor, MI).

Note that full information on the approval of the study protocol must also be provided in the manuscript.
