# Supplementary material for: Author Correction: Genome-wide association study of serum liver enzymes implicates diverse metabolic and liver pathology
Source: Nat Commun. 2023 Jun 8;14:3356. doi: 10.1038/s41467-023-38659-3 (PMC10250317; doi:10.1038/s41467-023-38659-3)
Supplement: Supplementary file 8 — Incorrect Supplementary Information [file 41467_2023_38659_MOESM8_ESM.pdf]

Supplementary Information:

Genome-wide association study of serum liver enzymes implicates diverse metabolic and liver pathology

Table of Contents

Supplementary Table 1: Descriptive summary of phenotypes in UK BioBank 2

Supplementary Table 2: Genomic control parameters 3

Supplementary Table 3: Alanine aminotransferase-altering alleles with heterogeneity between UK BioBank & BioBank Japan 4

Supplementary Table 4: Aspartate aminotransferase-altering alleles with heterogeneity between UK BioBank & BioBank Japan 5

Supplementary Table 5: Alkaline phosphatase-altering alleles with heterogeneity between UK BioBank & BioBank Japan 6

Supplementary Table 6: Alanine aminotransferase-altering alleles: BioBank Japan only 7

Supplementary Table 7: Aspartate aminotransferase-altering alleles: BioBank Japan only 8

Supplementary Table 8: Alanine aminotransferase-altering alleles with heterogeneity between men and women in UK BioBank 9

Supplementary Table 9: Aspartate aminotransferase-altering alleles with heterogeneity between men and women in UK BioBank 10

Supplementary Table 10: Alkaline phosphatase-altering alleles with heterogeneity between men and women in UK BioBank 11

Supplementary Table 11: Phenome-wide association studies of alanine aminotransferase-increasing alleles 12

Supplementary Table 12: Phenome-wide association studies of aspartate aminotransferase-increasing alleles 14

Supplementary Table 13: Phenome-wide association studies of alkaline phosphatase-increasing alleles 16

Supplementary Table 14: Latent causal variable analysis of alanine aminotransferase and metabolic traits 17

Supplementary Table 15: Latent causal variable analysis of aspartate aminotransferase and metabolic traits 18

Supplementary Table 16: Latent causal variable analysis of alkaline phosphatase and metabolic traits 19

Supplementary Table 17: Effect of all liver enzyme-increasing alleles on primary biliary cholangitis 20

Supplementary Table 18: Effects of polygenic risk scores on cirrhosis and hepatic steatosis in Michigan Genomics Initiative 21

Supplementary Figure 1: Quantile-quantile plots 22

Supplementary Figure 2: Effects of liver enzyme-increasing variants on metabolic traits. 23

Supplementary Figure 3: Effects of liver enzyme-increasing variants on serum/plasma metabolites. 24

Supplementary Figure 4: Associations between aspartate aminotransferase polygenic risk score and cirrhosis and steatosis. 25

Supplementary References 26

Supplementary Table 1: Descriptive summary of phenotypes in UK BioBank

Variable	Value	N
Female	54%	390812
Age (years)	67 (8.0)	390812
Alanine aminotransferase (U/L)	24 (14)	390812
Aspartate aminotransferase (U/L)	24 (11)	389565
Alkaline phosphatase (U/L)	84 (26)	390964
Triglycerides (mmol/L)	1.5 (1.0)	390616
High-density lipoprotein (mmol/L)	1.5 (0.38)	358767
Low-density lipoprotein (mmol/L)	3.7 (0.82)	321191
Systolic blood pressure (mmHg)	145 (19)	176321
Diastolic blood pressure (mmHg)	86 (11)	176322
Body mass index (kg/m2)	27 (4.8)	407545
Glucose (mmol/L)	5.1 (1.2)	358536
Waist-to-hip ratio (cm/cm)	0.87 (0.09)	407545

Values are expressed as mean (standard deviation) or percentage.

Supplementary Table 2: Genomic control parameters

Trait	UK BioBank		BioBank Japan		Meta-analysis
	Lambda_GC	Intercept	Lambda_GC	Intercept	Lambda_GC
Alanine aminotransferase	1.44	1.26	1.13	1.02	1.03
Aspartate aminotransferase	1.47	1.31	1.13	1.01	1.03
Alkaline phosphatase	1.71	1.54	1.15	1.06	1.03

Genomic control parameters (lambda_GC) and intercept estimates for the UK BioBank, BioBank Japan, and the meta-analysis. Lambda-GC estimates are from METAL and intercept from LDpred.

Supplementary Table 3: Alanine aminotransferase-altering alleles with heterogeneity between UK BioBank & BioBank Japan

CHR:POS	Variant	EA	OA	UK BioBank			BioBank Japan			Gene annotation	Nearest coding genes	P_het	Dir
				EAF	Beta	P	EAF	Beta	P				
10:101912064	rs2862954	T	C	0.50	0.071	2.41e-194	0.96	0.026	6.67e-03	ERLIN1 (e)	ERLIN1	4.34e-25	++
19:19379549	rs58542926	T	C	0.08	0.099	1.72e-106	0.08	0.034	9.20e-06	TM6SF2 (e)	TM6SF2	1.64e-08	++
4:146821410	rs4835265	A	C	0.16	0.064	1.21e-84	0.36	0.022	3.24e-08	ZNF827 (i)	ZNF827	1.88e-04	++
11:93870338	rs7117339	C	T	0.88	0.071	7.00e-80	0.95	0.038	3.45e-05	PANX1 (i)	PANX1	3.81e-06	++
1:16505320	rs1497406	G	A	0.58	0.046	1.38e-79	0.79	0.020	4.07e-05	EPHA2, ARHGEF19 (inter)	EPHA2, ARHGEF19	3.53e-06	++
19:45411941	rs429358	T	C	0.84	0.051	7.16e-54	0.90	0.016	1.03e-02	APOE (e)	APOE	9.12e-06	++
8:9185146	rs2126259	T	C	0.10	0.059	1.08e-50	0.01	0.055	3.68e-03	LOC157273 (i)	PPP1R3B, TNKS	7.51e-05	++
22:36545137	rs132642	T	A	0.83	0.051	7.52e-57	0.99	0.036	1.39e-01	APOL3 (UTR)	APOL3	3.74e-08	++
1:220970028	rs2642438	G	A	0.70	0.040	1.75e-52	0.83	0.008	1.37e-01	MARC1 (e)	C1orf115, HLX	1.73e-07	++
1:155106697	rs12904	G	A	0.59	0.035	7.71e-46	0.09	0.018	7.30e-03	EFNA1 (UTR)	EFNA1	1.35e-04	++
10:70985267	rs2394529	C	G	0.70	0.036	1.19e-42	0.18	0.009	7.17e-02	LOC101928994 (i)	HKDC1	1.61e-05	++
2:169834370	rs72623176	A	G	0.04	0.047	4.92e-14	0.33	0.045	1.22e-28	ABCB11 (i)	ABCB11	5.10e-09	++
8:145955007	rs2467663	T	C	0.66	0.034	3.90e-41	0.31	0.006	1.63e-01	ZNF251 (i)	ZNF251	5.77e-06	++
2:227112754	rs2943654	T	C	0.65	0.031	2.59e-34	0.91	0.006	3.39e-01	LOC646736, MIR5702 (inter)	NYAP2, IRS1	1.20e-05	++
8:10571491	rs4484649	C	A	0.40	0.029	5.44e-32	0.71	0.005	2.81e-01	C8orf74, SOX7 (inter)	C8orf74, SOX7	4.26e-05	++
2:165557318	rs6712203	C	T	0.63	0.028	7.71e-29	0.91	0.006	3.62e-01	COBLL1 (i)	COBLL1	7.42e-05	++
19:41333284	rs11878604	T	C	0.93	0.026	2.02e-08	0.61	0.036	6.94e-20	CYP2T1P, CYP2A6 (inter)	EGLN2, CYP2A6	4.37e-07	++
2:211540507	rs1047891	A	C	0.32	0.012	7.65e-06	0.15	0.049	6.46e-18	CPS1 (e)	CPS1	3.04e-07	++
1:150479901	rs1815544	C	T	0.60	0.024	2.22e-22	0.72	0.001	8.47e-01	TARS2 (UTR)	TARS2	7.82e-05	++
16:53806453	rs56094641	G	A	0.40	0.021	1.00e-17	0.20	-0.002	6.54e-01	FTO (i)	FTO	6.80e-05	+-

CHR:POS, chromosome:position. EA, effect allele. OA, other allele. EAF, effect allele frequency. P_het, P value for heterogeneity between UK BioBank and BioBank Japan. Gene tags: (e) exonic, (i) intronic, (u) upstream, (d) downstream, (inter) intergenic, (UTR) untranslated region. Dir, direction: + indicates that the variant increases the liver enzyme while - indicates that the variant decreases the liver enzyme. Direction of effect in UK BioBank is shown first, followed by effect in BioBank Japan.

Supplementary Table 4: Aspartate aminotransferase-altering alleles with heterogeneity between UK BioBank & BioBank Japan

CHR:POS	Variant	EA	OA	UK BioBank			BioBank Japan			Gene annotation	Nearest coding genes	P_het	Dir
				EAF	Beta	P	EAF	Beta	P				
10:101912064	rs2862954	T	C	0.50	0.056	2.60e-113	0.96	0.039	3.29e-05	ERLIN1 (e)	ERLIN1	1.13e-09	++
19:19379549	rs58542926	T	C	0.08	0.076	3.73e-59	0.08	0.029	1.38e-04	TM6SF2 (e)	TM6SF2	2.25e-04	++
3:58380465	rs11714574	A	T	0.59	0.045	2.43e-71	0.24	0.005	2.70e-01	PXK (i)	PXK	4.96e-11	++
1:16510894	rs36086195	T	C	0.58	0.041	8.37e-59	0.77	0.017	4.72e-04	EPHA2, ARHGEF19 (inter)	EPHA2, ARHGEF19	8.72e-05	++
8:8661681	rs12544992	G	C	0.47	0.033	1.91e-39	0.72	0.006	1.74e-01	MFHAS1 (i)	MFHAS1	1.13e-05	++
11:116648917	rs964184	G	C	0.13	0.043	8.54e-32	0.28	0.006	1.75e-01	ZPR1 (UTR)	ZPR1	1.42e-04	++
2:113841030	rs6734238	A	G	0.60	0.028	1.11e-28	0.95	0.003	6.98e-01	IL1F10, IL1RN (inter)	IL1F10, IL1RN	1.48e-05	++
10:70985267	rs2394529	C	G	0.70	0.029	5.40e-26	0.18	0.002	7.28e-01	LOC101928994 (i)	HKDC1	3.69e-05	++
22:36545137	rs132642	T	A	0.83	0.035	9.13e-27	0.99	-0.008	7.40e-01	APOL3 (UTR)	APOL3	2.06e-06	+-
1:66141502	rs1938500	T	G	0.19	0.032	2.69e-23	0.76	0.001	8.87e-01	LEPR, PDE4B (inter)	LEPR, PDE4B	5.30e-05	++
10:18263873	rs508196	C	T	0.56	0.088	1.72e-270	0.45	0.015	1.12e-04	SLC39A12 (i)	SLC39A12	6.81e-31	++
6:32582601	rs34656207	T	C	0.36	0.071	1.86e-159	0.46	0.029	6.04e-12	HLA-DRB1, HLA-DQA1 (inter)	HLA-DRB1, HLA-DQA1	1.44e-08	++
9:136141870	rs2519093	C	T	0.81	0.055	1.34e-66	0.72	0.014	6.32e-04	ABO (i)	ABO	9.19e-06	++
16:58770897	rs11643959	T	G	0.91	0.081	1.13e-75	0.99	0.011	5.96e-01	GOT2, APOOP5 (inter)	GOT2, CDH8	3.67e-13	++

CHR:POS, chromosome:position. EA, effect allele. OA, other allele. EAF, effect allele frequency. P_het, P value for heterogeneity between UK BioBank and BioBank Japan. Gene tags: (e) exonic, (i) intronic, (u) upstream, (d) downstream, (inter) intergenic, (UTR) untranslated region. Dir, direction: + indicates that the variant increases the liver enzyme while - indicates that the variant decreases the liver enzyme. Direction of effect in UK BioBank is shown first, followed by effect in BioBank Japan.

Supplementary Table 5: Alkaline phosphatase-altering alleles with heterogeneity between UK BioBank & BioBank Japan

CHR:POS	Variant	EA	OA	UK BioBank			BioBank Japan			Gene annotation	Nearest coding genes	P_het	Dir
				EAF	Beta	P	EAF	Beta	P				
1:21895061	rs1256330	T	C	0.31	0.108	1.59E-298	0.23	0.124	2.84E-124	ALPL (i)	ALPL	3.87e-11	++
10:65350383	rs10822186	G	A	0.49	0.102	<1e-300	0.49	0.039	6.35E-19	REEP3 (i)	REEP3	2.10e-09	++
12:121423659	rs9738226	A	G	0.38	0.086	1.82E-207	0.48	0.021	1.57E-06	HNF1A (i)	HNF1A	7.05e-12	++
2:27730940	rs1260326	T	C	0.39	0.071	4.02E-146	0.56	0.014	1.12E-03	GCKR (e)	GCKR	1.84e-10	++
19:54677189	rs8736	T	C	0.44	0.066	4.58E-131	0.22	0.019	2.61E-04	MBOAT7 (UTR)	MBOAT7	2.67e-08	++
11:296255	rs12277152	A	G	0.05	0.115	1.25E-73	0.28	0.064	2.72E-38	PGGHG (d)	PGGHG, IFITM5	1.62e-05	++
19:19379549	rs58542926	C	T	0.92	0.127	3.84E-136	0.92	0.017	4.92E-02	TM6SF2 (e)	TM6SF2	1.06e-12	++
19:45421877	rs484195	A	G	0.38	0.066	4.51E-120	0.59	0.018	5.09E-04	APOC1 (i)	APOC1	9.65e-08	++
11:61592362	rs174566	G	A	0.35	0.063	3.57E-109	0.39	0.020	7.98E-06	FADS2 (i)	FADS2	3.69e-05	++
8:10665444	rs6601527	C	A	0.41	0.061	6.75E-108	0.66	0.011	1.74E-02	PINX1 (i)	PINX1	6.34e-09	++
2:169893419	rs2161037	G	A	0.45	0.056	5.33E-94	0.29	0.008	1.14E-01	ABCB11, DHRS9 (inter)	ABCB11, DHRS9	2.88e-09	++
15:58678720	rs261290	C	T	0.65	0.055	1.93E-82	0.57	0.010	1.90E-02	AQP9, LIPC (inter)	AQP9, LIPC	1.32e-06	++
4:100065509	rs1800759	T	G	0.39	0.052	1.61E-78	0.16	0.007	2.58E-01	LOC100507053 (i)	ADH4, ADH6	1.40e-08	++
16:72217113	rs7202323	G	T	0.23	0.051	1.11E-55	0.37	0.005	3.16E-01	PMFBP1, LINC01572 (inter)	PMFBP1, ZFHX3	2.50e-06	++
1:20894442	rs12137738	A	T	0.90	0.056	2.29E-35	0.99	0.000	9.86E-01	FAM43B, CDA (inter)	FAM43B, CDA	1.15e-05	+-
9:136140462	rs1633513	C	T	0.27	0.118	<1e-300	0.27	0.137	1.06E-173	ABO (i)	ABO	3.28e-22	++
8:9194978	rs13274716	C	T	0.12	0.162	<1e-300	0.01	0.106	1.02E-06	LOC157273, TNKS (inter)	PPP1R3B, TNKS	1.81e-21	++
8:126500031	rs28601761	C	G	0.58	0.100	1.35E-286	0.84	0.021	7.51E-04	TRIB1, LINC00861 (inter)	TRIB1, POU5F1B	2.32e-23	++
20:25296970	rs6083799	T	G	0.56	0.067	1.92E-134	0.08	0.023	8.19E-03	ABHD12 (i)	ABHD12	7.68e-11	++
14:24871926	rs11621792	C	T	0.55	0.044	1.17E-58	0.95	0.006	5.29E-01	NYNRIN (i)	NYNRIN	2.36e-07	++
4:69326683	rs4694077	C	T	0.68	0.042	1.26E-47	0.91	0.011	1.98E-01	TMPRSS11E (i)	TMPRSS11E	5.27e-05	++
12:53727545	rs930900	C	T	0.69	0.037	2.56E-37	0.99	0.006	8.10E-01	SP7 (i)	SP7	1.71e-05	++
12:4606168	rs2970818	T	A	0.90	0.050	3.06E-29	1.00	-0.011	8.02E-01	C12orf4 (i)	C12orf4	3.15e-05	+-
6:116418112	rs4946137	A	G	0.40	0.026	1.45E-20	0.69	-0.003	4.67E-01	FRK, NT5DC1 (inter)	FRK, NT5DC1	1.06e-04	+-

CHR:POS, chromosome:position. EA, effect allele. OA, other allele. EAF, effect allele frequency. P_het, P value for heterogeneity between UK BioBank and BioBank Japan. Gene tags: (e) exonic, (i) intronic, (u) upstream, (d) downstream, (inter) intergenic, (UTR) untranslated region. Dir, direction: + indicates that the variant increases the liver enzyme while - indicates that the variant decreases the liver enzyme. Direction of effect in UK BioBank is shown first, followed by effect in BioBank Japan.

Supplementary Table 6: Alanine aminotransferase-altering alleles: BioBank Japan only

CHR:POS	Variant	EA	OA	BioBank Japan			UK BioBank			Gene annotation	Nearest coding genes
				EAF	Beta	P	EAF	Beta	P		
12:113548243	rs1902955	T	C	0.81	0.029	1.90e-09	0.61	0.0024	3.31e-01	RASAL1 (i)	RASAL1
6:33860843	rs185355701	T	G	0.055	0.047	3.43e-08	0.057	0.0031	5.48e-01	LINC01016 (i)	MLN, GRM4

CHR:POS, chromosome:position. EA, effect allele. OA, other allele. EAF, effect allele frequency. Gene tags: (e) exonic, (i) intronic, (u) upstream, (d) downstream, (inter) intergenic, (UTR) untranslated region.

Supplementary Table 7: Aspartate aminotransferase-altering alleles: BioBank Japan only

				BioBank Japan			UK BioBank				
CHR:POS	Variant	EA	OA	EAF	Beta	P	EAF	Beta	P	Gene annotation	Nearest coding genes
19:41333284	rs11878604	T	C	0.61	0.03	2.14e-14	0.93	0.0074	1.29e-01	CYP2T1P, CYP2A6 (inter)	EGLN2, CYP2A6
7:80174361	rs139761834	T	C	0.95	0.08	3.45e-14				GNAT3, CD36 (inter)	GNAT3, CD36
4:79619199	rs75759936	C	A	0.82	0.032	1.58e-08				LINC01094, BMP2K (inter)	ANXA3, BMP2K
7:50258479	rs4598207	A	T	0.57	0.022	2.82e-08	0.69	-0.0035	1.93e-01	C7orf72, IKZF1 (inter)	ZPBP, IKZF1
12:110069190	rs11067592	G	T	0.92	0.04	3.49e-08				MVK, FAM222A (inter)	MVK, FAM222A

CHR:POS, chromosome:position. EA, effect allele. OA, other allele. EAF, effect allele frequency. Gene tags: (e) exonic, (i) intronic, (u) upstream, (d) downstream, (inter) intergenic, (UTR) untranslated region.

Supplementary Table 8: Alanine aminotransferase-altering alleles with heterogeneity between men and women in UK BioBank

CHR:POS	Variant	EA	OA	Male			Female			Gene annotation	Nearest coding genes	P_het	Dir
				EAF	Beta	P	EAF	Beta	P				
9:117146043	rs7041363	C	G	0.51	0.07	8.19E-88	0.51	0.049	3.17E-51	AKNA (i)	AKNA	1.09E-04	++
8:126482077	rs2954021	A	G	0.49	0.064	1.45E-74	0.49	0.035	5.05E-27	TRIB1, LINC00861 (inter)	TRIB1, FAM84B	5.45E-08	++
4:146821410	rs4835265	A	C	0.16	0.045	5.41E-21	0.16	0.075	1.68E-64	ZNF827 (i)	ZNF827	3.59E-05	++
1:155106697	rs12904	G	A	0.59	0.019	1.89E-07	0.59	0.046	5.12E-44	EFNA1 (UTR)	EFNA1	4.89E-07	++
10:70985267	rs2394529	C	G	0.70	0.012	1.37E-03	0.70	0.055	6.44E-54	LOC101928 994 (i)	HKDC1	3.19E-13	++
16:80497341	rs2865001 2	G	C	0.27	0.017	1.25E-05	0.27	0.041	7.13E-29	LOC102724 084 (i)	MAF, DYNLRB2	9.35E-05	++
16:83980529	rs4782568	C	G	0.55	0.035	2.01E-23	0.55	0.015	2.60E-06	MLYCD, OSGIN1 (inter)	MLYCD, OSGIN1	2.35E-04	++

CHR:POS, chromosome:position. EA, effect allele. OA, other allele. EAF, effect allele frequency. P_het, P value for heterogeneity between men and women. Gene tags: (e) exonic, (i) intronic, (u) upstream, (d) downstream, (inter) intergenic, (UTR) untranslated region. Dir, direction: + indicates that the variant increases the liver enzyme while - indicates that the variant decreases the liver enzyme. Direction of effect in UK BioBank male is shown as effect in men followed by effect in women.

Supplementary Table 9: Aspartate aminotransferase-altering alleles with heterogeneity between men and women in UK BioBank

CHR:POS	Variant	EA	OA	Male			Female			Gene annotation	Nearest coding genes	P_het	Dir
				EAF	Beta	P	EAF	Beta	P				
1:155106697	rs12904	G	A	0.59	0.014	8.98E-05	0.59	0.040	8.16E-34	EFNA1 (UTR)	EFNA1	3.07E-06	++
11:116648917	rs964184	G	C	0.13	0.063	5.74E-33	0.13	0.022	2.69E-06	ZPR1 (UTR)	ZPR1	3.47E-07	++
10:70985267	rs2394529	C	G	0.70	0.0071	6.93E-02	0.70	0.044	2.69E-35	LOC101928994 (i)	HKDC1	5.12E-10	++
4:77416627	rs12500824	A	G	0.35	0.029	9.59E-15	0.35	0.0056	9.53E-02	SHROOM3 (i)	SHROOM3	4.00E-05	++
19:55824332	rs7246479	G	T	0.51	0.002	5.82E-01	0.51	0.024	3.02E-13	TMEM150B (e)	TMEM150B	6.53E-05	++

CHR:POS, chromosome:position. EA, effect allele. OA, other allele. EAF, effect allele frequency. P_het, P value for heterogeneity between men and women. Gene tags: (e) exonic, (i) intronic, (u) upstream, (d) downstream, (inter) intergenic, (UTR) untranslated region. Dir, direction: + indicates that the variant increases the liver enzyme while - indicates that the variant decreases the liver enzyme. Direction of effect in UK BioBank male is shown as effect in men followed by effect in women.

Supplementary Table 10: Alkaline phosphatase-altering alleles with heterogeneity between men and women in UK BioBank

CHR:POS	Variant	EA	OA	Male			Female			Gene annotation	Nearest coding genes	P_het	Dir
				EAF	Beta	P	EAF	Beta	P				
19:19379549	rs58542926	C	T	0.92	0.15	3.35E-96	0.92	0.084	3.27E-42	TM6SF2 (e)	TM6SF2	1.52E-08	++
9:136140462	rs1633513	C	T	0.26	0.13	9.70E-205	0.27	0.10	1.00E-162	ABO (i)	ABO	1.75E-05	++
19:49164952	rs281392	A	G	0.33	0.11	2.40E-164	0.33	0.081	3.00E-117	NTN5 (e)	NTN5	6.07E-06	++
8:126500031	rs28601761	C	G	0.58	0.12	1.80E-204	0.58	0.067	4.23E-88	TRIB1, LINC00861 (inter)	TRIB1, FAM84B	7.47E-17	++
20:25296970	rs6083799	T	G	0.56	0.076	2.35E-90	0.56	0.046	3.68E-45	ABHD12 (i)	ABHD12	6.77E-07	++
4:69326683	rs4694077	C	T	0.68	0.053	2.09E-38	0.68	0.023	6.23E-11	TMPRSS11E (i)	TMPRSS11E	4.13E-06	++
6:25783315	rs3923	T	C	0.42	0.039	7.19E-25	0.42	0.015	3.72E-06	SLC17A1 (UTR)	SLC17A1	7.14E-05	++
5:88364958	rs6886306	T	C	0.47	0.036	6.88E-22	0.47	0.014	3.14E-05	MEF2C-AS1 (i)	MEF2C, CETN3	1.48E-04	++

CHR:POS, chromosome:position. EA, effect allele. OA, other allele. EAF, effect allele frequency. P_het, P value for heterogeneity between men and women. Gene tags: (e) exonic, (i) intronic, (u) upstream, (d) downstream, (inter) intergenic, (UTR) untranslated region. Dir, direction: + indicates that the variant increases the liver enzyme while - indicates that the variant decreases the liver enzyme. Direction of effect in UK BioBank male is shown as effect in men followed by effect in women.

Supplementary Table 11: Phenome-wide association studies of alanine aminotransferase-increasing alleles

Variant	Trait	EA	OA	EAF	Beta	P	Gene annotation
rs1277930	E78 Disorders of lipoprotein metabolism and other lipidaemias	A	G	0.81	0.01	1.22e-55	PSRC1 (d)
rs1002436	I10 Essential (primary) hypertension	G	A	0.56	-0.0041	3.44e-08	PKN2-AS1 (i)
rs1277930	I20 Angina pectoris	A	G	0.81	0.0042	2.46e-17	PSRC1 (d)
rs1277930	I21 Acute myocardial infarction	A	G	0.81	0.002	2.06e-09	PSRC1 (d)
rs1277930	I25 Chronic ischaemic heart disease	A	G	0.81	0.0059	4.30e-25	PSRC1 (d)
rs1538742	K42 Umbilical hernia	A	C	0.54	-0.0015	2.04e-13	LOC102723886 (i)
rs6712203	E11 Non-insulin-dependent diabetes mellitus	C	T	0.7	0.0031	1.51e-13	COBLL1 (i)
rs2943654	E11 Non-insulin-dependent diabetes mellitus	T	C	0.71	0.0037	4.25e-18	LOC646736, MIR5702 (inter)
rs11887534	E78 Disorders of lipoprotein metabolism and other lipidaemias	C	G	0.051	-0.0083	2.03e-13	ABCG8 (e)
rs11887534	K80 Cholelithiasis	C	G	0.051	0.026	7.59e-257	ABCG8 (e)
rs11887534	K81 Cholecystitis	C	G	0.051	0.0041	5.93e-34	ABCG8 (e)
rs11887534	K82 Other diseases of gallbladder	C	G	0.051	0.0034	1.92e-35	ABCG8 (e)
rs11887534	K83 Other diseases of biliary tract	C	G	0.051	0.0022	4.36e-14	ABCG8 (e)
rs645040	I10 Essential (primary) hypertension	T	G	0.79	0.005	1.02e-08	MSL2, PCCB (inter)
rs645040	I21 Acute myocardial infarction	T	G	0.79	0.0019	2.87e-08	MSL2, PCCB (inter)
rs78900599	K80 Cholelithiasis	A	G	0.92	0.0048	3.51e-11	TM4SF1-AS1, TM4SF4 (inter)
rs40270	E11 Non-insulin-dependent diabetes mellitus	C	A	0.61	0.0028	1.07e-08	LINC01948, C5orf67 (inter)
rs1468615	K80 Cholelithiasis	T	C	0.8	0.0036	2.60e-13	ABCB4 (i)
rs2954021	E78 Disorders of lipoprotein metabolism and other lipidaemias	A	G	0.48	0.0068	8.90e-35	TRIB1, LINC00861 (inter)
rs4484649	I10 Essential (primary) hypertension	C	A	0.47	0.0055	2.98e-13	C8orf74, SOX7 (inter)
rs2954021	I25 Chronic ischaemic heart disease	A	G	0.48	0.0028	3.74e-09	TRIB1, LINC00861 (inter)
rs687621	E78 Disorders of lipoprotein metabolism and other lipidaemias	G	A	0.36	0.0034	2.00e-08	ABO (i)
rs687621	I26 Pulmonary embolism	G	A	0.36	0.0028	3.71e-42	ABO (i)
rs687621	I80 Phlebitis and thrombophlebitis	G	A	0.36	0.0036	4.15e-62	ABO (i)
rs687621	I84 Haemorrhoids	G	A	0.36	-0.0041	1.63e-15	ABO (i)
rs687621	K57 Diverticular disease of intestine	G	A	0.36	-0.0039	2.73e-13	ABO (i)
rs687621	M79 Other soft tissue disorders, not elsewhere classified	G	A	0.36	0.0024	4.19e-10	ABO (i)
rs1051713	K80 Cholelithiasis	C	T	0.86	-0.0038	1.23e-14	ALOX5 (i)
rs4766462	E03 Other hypothyroidism	A	T	0.64	0.0029	1.35e-10	SH2B3 (i)
rs4766462	I10 Essential (primary) hypertension	A	T	0.64	0.0058	1.07e-10	SH2B3 (i)
rs864899	I83 Varicose veins of lower extremities	G	A	0.52	-0.002	2.38e-09	ATF1, TMPRSS12 (inter)
rs11061602	M16 Coxarthrosis [arthrosis of hip]	T	G	0.51	-0.0019	1.80e-08	MLXIP (i)
rs56094641	E11 Non-insulin-dependent diabetes mellitus	G	A	0.35	0.0044	6.85e-26	FTO (i)
rs56094641	E66 Obesity	G	A	0.35	0.004	3.00e-33	FTO (i)
rs56094641	I10 Essential (primary) hypertension	G	A	0.35	0.0048	9.48e-11	FTO (i)
rs17138478	K80 Cholelithiasis	C	A	0.85	-0.0031	4.72e-08	HNF1B (i)
rs429358	G30-G32 Other degenerative diseases of the nervous system	T	C	0.86	-0.0022	4.15e-54	APOE (e)

rs58542926	E78 Disorders of lipoprotein metabolism and other lipidaemias	T	C	0.076	-0.0088	9.56e-17	TM6SF2 (e)
rs429358	E78 Disorders of lipoprotein metabolism and other lipidaemias	T	C	0.86	-0.014	1.81e-74	APOE (e)
rs429358	F05 Delirium, not induced by alcohol and other psychoactive substances	T	C	0.86	-0.00076	1.86e-12	APOE (e)
rs429358	I20 Angina pectoris	T	C	0.86	-0.0046	1.12e-15	APOE (e)
rs429358	I21 Acute myocardial infarction	T	C	0.86	-0.0024	7.00e-10	APOE (e)
rs429358	I25 Chronic ischaemic heart disease	T	C	0.86	-0.0059	2.49e-19	APOE (e)
rs58542926	K76 Other diseases of liver	T	C	0.076	0.0021	8.23e-09	TM6SF2 (e)
rs7599	K80 Cholelithiasis	A	G	0.33	0.0022	6.85e-09	TMEM147 (UTR)
rs738409	I85 Oesophageal varices	G	C	0.28	0.00068	2.97e-14	PNPLA3 (e)
rs738409	K70 Alcoholic liver disease	G	C	0.28	0.00088	1.98e-14	PNPLA3 (e)
rs738409	K74 Fibrosis and cirrhosis of liver	G	C	0.28	0.00076	2.25e-11	PNPLA3 (e)
rs738409	K76 Other diseases of liver	G	C	0.28	0.0022	4.77e-22	PNPLA3 (e)

CHR:POS, chromosome:position. EA, effect allele. OA, other allele. EAF, effect allele frequency. Traits are represented as International Classification of Diseases code followed by disease name. Gene tags: (e) exonic, (i) intronic, (u) upstream, (d) downstream, (inter) intergenic, (UTR) untranslated region.

Supplementary Table 12: Phenome-wide association studies of aspartate aminotransferase-increasing alleles

Variant	Trait	EA	OA	EAF	Beta	P	Gene annotation
rs1002436	I10 Essential (primary) hypertension	G	A	0.56	-0.0041	3.44e-08	PKN2-AS1 (i)
rs6547692	E11 Non-insulin-dependent diabetes mellitus	G	A	0.47	-0.0022	4.53e-08	GCKR (i)
rs2943654	E11 Non-insulin-dependent diabetes mellitus	T	C	0.71	0.0037	4.25e-18	LOC646736, MIR5702 (inter)
rs6547692	E78 Disorders of lipoprotein metabolism and other lipidaemias	G	A	0.47	0.0041	2.46e-13	GCKR (i)
rs6547692	K80 Cholelithiasis	G	A	0.47	-0.0024	4.74e-10	GCKR (i)
rs6547692	M10 Gout	G	A	0.47	0.0012	6.31e-11	GCKR (i)
rs2594973	I10 Essential (primary) hypertension	C	G	0.37	-0.005	6.75e-11	ATG7 (i)
rs6894249	J45 Asthma	A	G	0.55	-0.0032	1.66e-10	C5orf56 (i)
rs13212562	E03 Other hypothyroidism	A	G	0.89	-0.0034	8.80e-10	VN1R10P, ZNF204P (inter)
rs13212562	E05 Thyrotoxicosis [hyperthyroidism]	A	G	0.89	-0.0013	5.10e-10	VN1R10P, ZNF204P (inter)
rs13212562	E10 Insulin-dependent diabetes mellitus	A	G	0.89	-0.0015	4.41e-09	VN1R10P, ZNF204P (inter)
rs6916318	I83 Varicose veins of lower extremities	T	A	0.52	-0.0018	3.86e-08	MIR588, RSPO3 (inter)
rs13212562	K40 Inguinal hernia	A	G	0.89	0.0035	1.65e-09	VN1R10P, ZNF204P (inter)
rs9372475	K40 Inguinal hernia	T	C	0.35	0.0023	1.36e-08	RFX6, VGLL2 (inter)
rs13212562	K90 Intestinal malabsorption	A	G	0.89	-0.0059	6.94e-152	VN1R10P, ZNF204P (inter)
rs13212562	N40 Hyperplasia of prostate	A	G	0.89	0.0077	5.15e-13	VN1R10P, ZNF204P (inter)
rs2954027	E78 Disorders of lipoprotein metabolism and other lipidaemias	T	A	0.52	0.0068	6.26e-34	TRIB1, LINC00861 (inter)
rs12544992	I10 Essential (primary) hypertension	G	C	0.53	0.0047	2.16e-10	MFHAS1 (i)
rs4841436	I10 Essential (primary) hypertension	C	A	0.48	0.0061	3.73e-16	LOC10272331 3 (i)
rs2954027	I25 Chronic ischaemic heart disease	T	A	0.52	0.0028	2.28e-09	TRIB1, LINC00861 (inter)
rs113895159	K80 Cholelithiasis	T	C	0.59	-0.0025	5.94e-10	SDCBP (i)
rs1800978	E78 Disorders of lipoprotein metabolism and other lipidaemias	C	G	0.85	0.0052	1.08e-09	ABCA1 (UTR)
rs2519093	E78 Disorders of lipoprotein metabolism and other lipidaemias	C	T	0.79	-0.0057	2.63e-15	ABO (i)
rs2519093	I26 Pulmonary embolism	C	T	0.79	-0.0037	2.29e-49	ABO (i)
rs2519093	I80 Phlebitis and thrombophlebitis	C	T	0.79	-0.0041	3.52e-57	ABO (i)
rs2519093	I84 Haemorrhoids	C	T	0.79	0.0036	6.03e-09	ABO (i)
rs2519093	K57 Diverticular disease of intestine	C	T	0.79	0.0036	2.51e-08	ABO (i)
rs2519093	M79 Other soft tissue disorders, not elsewhere classified	C	T	0.79	-0.0028	7.57e-10	ABO (i)
rs964184	E78 Disorders of lipoprotein metabolism and other lipidaemias	G	C	0.17	0.0096	1.00e-31	ZPR1 (UTR)
rs705699	E03 Other hypothyroidism	G	A	0.63	-0.0022	1.57e-09	RAB5B (i)
rs705699	J33 Nasal polyp	G	A	0.63	-0.001	4.13e-08	RAB5B (i)
rs705699	J45 Asthma	G	A	0.63	-0.0031	5.48e-10	RAB5B (i)
rs217184	E78 Disorders of lipoprotein metabolism and other lipidaemias	T	C	0.8	0.0039	3.83e-08	HPR (i)
rs17138478	K80 Cholelithiasis	C	A	0.85	-0.0031	4.72e-08	HNF1B (i)
rs58542926	E78 Disorders of lipoprotein metabolism and other lipidaemias	T	C	0.076	-0.0088	9.56e-17	TM6SF2 (e)

rs439401	E78 Disorders of lipoprotein metabolism and other lipidaemias	C	T	0.58	0.0046	3.36e-15	APOE, APOC1 (inter)
rs11882796	E78 Disorders of lipoprotein metabolism and other lipidaemias	T	A	0.42	0.0038	7.62e-12	RASIP1 (i)
rs11882796	I10 Essential (primary) hypertension	T	A	0.42	0.0045	5.56e-10	RASIP1 (i)
rs58542926	K76 Other diseases of liver	T	C	0.076	0.0021	8.23e-09	TM6SF2 (e)
rs11882796	K80 Cholelithiasis	T	A	0.42	0.0029	3.12e-14	RASIP1 (i)
rs738409	I85 Oesophageal varices	G	C	0.28	0.00068	2.97e-14	PNPLA3 (e)
rs738409	K70 Alcoholic liver disease	G	C	0.28	0.00088	1.98e-14	PNPLA3 (e)
rs738409	K74 Fibrosis and cirrhosis of liver	G	C	0.28	0.00076	2.25e-11	PNPLA3 (e)
rs738409	K76 Other diseases of liver	G	C	0.28	0.0022	4.77e-22	PNPLA3 (e)

CHR:POS, chromosome:position. EA, effect allele. OA, other allele. EAF, effect allele frequency. Traits are represented as International Classification of Diseases code followed by disease name. Gene tags: (e) exonic, (i) intronic, (u) upstream, (d) downstream, (inter) intergenic, (UTR) untranslated region.

Supplementary Table 13: Phenome-wide association studies of alkaline phosphatase-increasing alleles

Variant	Trait	EA	OA	EAF	Beta	P	Gene annotation
rs1260326	E11 Non-insulin-dependent diabetes mellitus	T	C	0.43	-0.0027	1.88e-10	GCKR (e)
rs1260326	E78 Disorders of lipoprotein metabolism and other lipidaemias	T	C	0.43	0.0048	3.09e-17	GCKR (e)
rs1260326	K80 Cholelithiasis	T	C	0.43	-0.0026	9.37e-12	GCKR (e)
rs1260326	M10 Gout	T	C	0.43	0.0014	2.81e-14	GCKR (e)
rs687339	I10 Essential (primary) hypertension	T	C	0.78	0.0049	2.38e-08	MSL2, PCCB (inter)
rs687339	I21 Acute myocardial infarction	T	C	0.78	0.0019	2.56e-08	MSL2, PCCB (inter)
rs12633863	K80 Cholelithiasis	A	G	0.54	-0.0033	2.82e-18	TM4SF4 (i)
rs2290846	K80 Cholelithiasis	G	A	0.71	-0.004	1.16e-21	LRBA (e)
rs2728102	M10 Gout	T	C	0.79	-0.0027	9.96e-24	PKD2 (i)
rs3923	E83 Disorders of mineral metabolism	T	C	0.47	0.0023	4.41e-52	SLC17A1 (UTR)
rs764284	E83 Disorders of mineral metabolism	G	A	0.56	0.001	8.38e-12	VN1R10P, ZNF204P (inter)
rs6912283	I10 Essential (primary) hypertension	A	G	0.58	-0.0045	8.04e-10	ZNF318, ABCC10 (inter)
rs3923	K90 Intestinal malabsorption	T	C	0.47	0.0015	6.60e-23	SLC17A1 (UTR)
rs764284	K90 Intestinal malabsorption	G	A	0.56	0.0016	2.96e-25	VN1R10P, ZNF204P (inter)
rs28601761	E78 Disorders of lipoprotein metabolism and other lipidaemias	C	G	0.63	0.0076	4.43e-40	TRIB1, LINC00861 (inter)
rs6601527	I10 Essential (primary) hypertension	C	A	0.46	0.0058	4.15e-15	PINX1 (i)
rs28601761	I25 Chronic ischaemic heart disease	C	G	0.63	0.0033	1.03e-11	TRIB1, LINC00861 (inter)
rs1633513	I80 Phlebitis and thrombophlebitis	C	T	0.27	-0.0018	1.38e-15	ABO (i)
rs10900229	K80 Cholelithiasis	C	T	0.78	-0.0036	2.13e-17	ZFAND4 (i)
rs10822186	K80 Cholelithiasis	G	A	0.49	0.0022	9.47e-09	REEP3 (i)
rs174566	J45 Asthma	G	A	0.36	-0.003	9.23e-09	FADS2 (i)
rs174566	K80 Cholelithiasis	G	A	0.36	0.0023	7.15e-09	FADS2 (i)
rs2073950	E03 Other hypothyroidism	C	T	0.79	0.0029	6.98e-11	ATXN2 (i)
rs2073950	I10 Essential (primary) hypertension	C	T	0.79	0.006	3.28e-11	ATXN2 (i)
rs9738226	I25 Chronic ischaemic heart disease	A	G	0.4	0.003	8.11e-10	HNF1A (i)
rs9738226	K80 Cholelithiasis	A	G	0.4	-0.0027	1.26e-12	HNF1A (i)
rs2941505	J45 Asthma	A	G	0.35	0.0032	2.39e-09	PGAP3 (i)
rs58542926	E78 Disorders of lipoprotein metabolism and other lipidaemias	C	T	0.92	0.0088	9.56e-17	TM6SF2 (e)
rs58542926	K76 Other diseases of liver	C	T	0.92	-0.0021	8.23e-09	TM6SF2 (e)
rs6088730	C44 Other malignant neoplasms of skin	C	G	0.57	-0.002	3.50e-08	EDEM2 (i)
rs17216707	N20 Calculus of kidney and ureter	C	T	0.17	-0.0018	9.04e-10	BCAS1, CYP24A1 (inter)
rs2836882	K51 Ulcerative colitis	G	A	0.75	0.0017	7.06e-16	LINC01700, PSMG1 (inter)

CHR:POS, chromosome:position. EA, effect allele. OA, other allele. EAF, effect allele frequency. Traits are represented as International Classification of Diseases code followed by disease name. Gene tags: (e) exonic, (i) intronic, (u) upstream, (d) downstream, (inter) intergenic, (UTR) untranslated region.

Supplementary Table 14: Latent causal variable analysis of alanine aminotransferase and metabolic traits

Trait	GCP	GCP Z score	Rho	Rho Z score	Heritability of metabolic trait (Z score)	Heritability of liver enzyme (Z score)
Triglycerides	0.65	2.86	0.18	1.04	10.08	12.71
High-density lipoprotein	0.53	2.25	-0.31	-5.61	9.26	12.72
Body mass index	0.65	2.48	0.40	6.05	26.27	12.71
Waist-hip-ratio	0.09	0.72	0.13	0.94	19.63	12.71
Fasting glucose	-0.26	-1.51	0.19	3.22	8.10	12.71
Systolic blood pressure	-0.20	-0.93	0.32	4.16	23.09	12.71
Diastolic blood pressure	-0.19	-0.90	0.29	3.57	22.83	12.71

GCP, genetic causality proportion. Significantly positive GCP implies that the metabolic trait is causal for the liver enzyme, and negative GCP implies that the liver enzyme is causal for the metabolic trait. Rho represents the estimated genetic correlation between the liver enzyme and the metabolic trait. SE, standard error.

Supplementary Table 15: Latent causal variable analysis of aspartate aminotransferase and metabolic traits

Trait	GCP	GCP Z score	Rho	Rho Z score	Heritability of metabolic trait (Z score)	Heritability of liver enzyme (Z score)
Triglycerides	0.24	0.76	0.02	0.10	10.08	11.69
High-density lipoprotein	0.34	1.78	-0.02	-0.34	9.26	11.69
Body mass index	0.37	0.97	0.17	1.87	26.27	11.69
Waist-hip-ratio	0.22	0.50	0.01	0.10	19.65	11.70
Fasting glucose	0.24	0.71	-0.01	-0.10	8.10	11.69
Systolic blood pressure	-0.46	-2.07	0.24	2.65	23.09	11.69
Diastolic blood pressure	-0.41	-1.87	0.19	1.98	22.83	11.69

GCP, genetic causality proportion. Significantly positive GCP implies that the metabolic trait is causal for the liver enzyme, and negative GCP implies that the liver enzyme is causal for the metabolic trait. Rho represents the estimated genetic correlation between the liver enzyme and the metabolic trait. SE, standard error.

Supplementary Table 16: Latent causal variable analysis of alkaline phosphatase and metabolic traits

Trait	GCP	GCP Z score	Rho	Rho Z score	Heritability of metabolic trait (Z score)	Heritability of liver enzyme (Z score)
Triglycerides	0.38	1.08	-0.04	-0.20	10.08	6.53
High-density lipoprotein	0.20	1.06	-0.08	-1.44	9.26	6.53
Body mass index	0.29	0.64	0.21	1.83	26.27	6.53
Waist-hip-ratio	-0.57	-2.15	-0.09	-0.55	19.63	6.53
Fasting glucose	-0.07	-0.09	0.07	1.07	8.10	6.53
Systolic blood pressure	-0.01	-0.02	0.24	2.19	23.09	6.55
Diastolic blood pressure	-0.01	-0.02	0.25	2.20	22.83	6.55

GCP, genetic causality proportion. Significantly positive GCP implies that the metabolic trait is causal for the liver enzyme, and negative GCP implies that the liver enzyme is causal for the metabolic trait. Rho represents the estimated genetic correlation between the liver enzyme and the metabolic trait. SE, standard error.

Supplementary Table 17: Effect of all liver enzyme-increasing alleles on primary biliary cholangitis

CHR:POS	Variant	EA	OA	EAF	Beta	P	Proxy	R2	Gene annotation
3:161823590	rs17236494	A (C)	T (T)	0.24	0.26	2.6e-01	rs4679904	0.85	*ARF7*
4:103947120	rs223454	A (A)	G (C)	0.52	0.21	2.1e-01	rs223420	0.98	*UBE2D3P*
4:103797685	rs179195	T (A)	C (G)	0.51	-0.22	-2.2e-01	rs228614	0.85	*MANBA*

CHR:POS, chromosome:position. EA, effect allele. OA, other allele. EAF, effect allele frequency. When liver enzyme-increasing variants were not themselves available, proxy variants were used. EA and OA of proxy variants are shown in parentheses. Gene tags: (e) exonic, (i) intronic, (u) upstream, (d) downstream, (inter) intergenic, (UTR) untranslated region.

Supplementary Table 18: Effects of polygenic risk scores on cirrhosis and hepatic steatosis in Michigan Genomics Initiative

	Cirrhosis		Hepatic steatosis	
Liver enzyme	Odds ratio	P value	Odds ratio	P value
Alanine aminotransferase	1.23 (1.17-1.30)	5.10E-15	1.17 (1.14-1.21)	9.60E-28
Asparate aminotransferase	1.10 (1.04-1.16)	5.10E-04	1.08 (1.05-1.11)	3.80E-08
Alkaline phosphatase	1.05 (0.99-1.10)	8.60E-02	1.02 (0.99-1.05)	1.20E-01

Effect of each rank unit of polygenic risk score for a specific liver enzyme on cirrhosis and hepatic steatosis. Effects are represented as odds ratio (95% confidence interval).

Supplementary Figure 1: Quantile-quantile plots

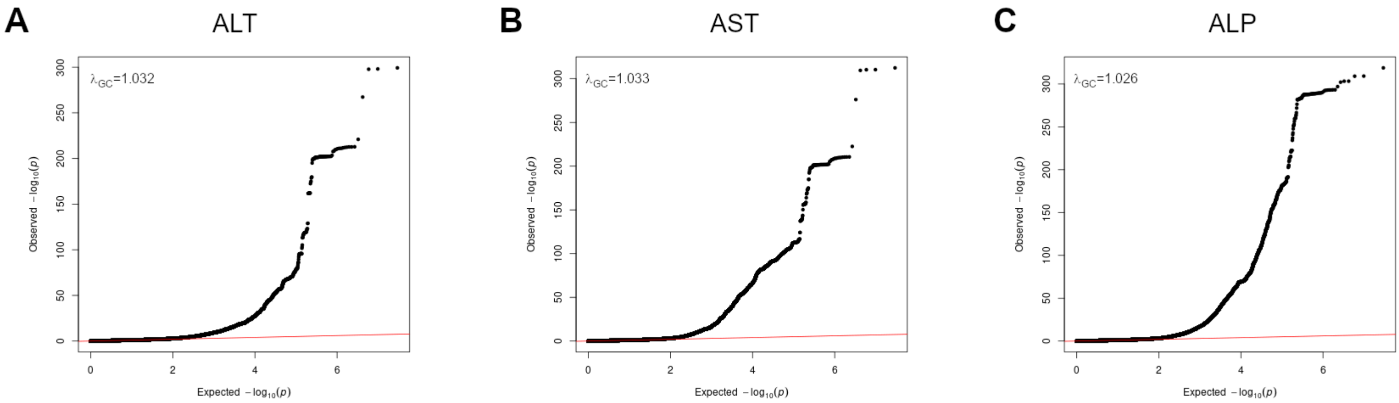

(A-C) Quantile-quantile plots for genetic variants affecting (A) alanine aminotransferase (ALT), (B) aspartate aminotransferase (AST), or (C) alkaline phosphatase (ALP).

Supplementary Figure 2: Effects of liver enzyme-increasing variants on metabolic traits.

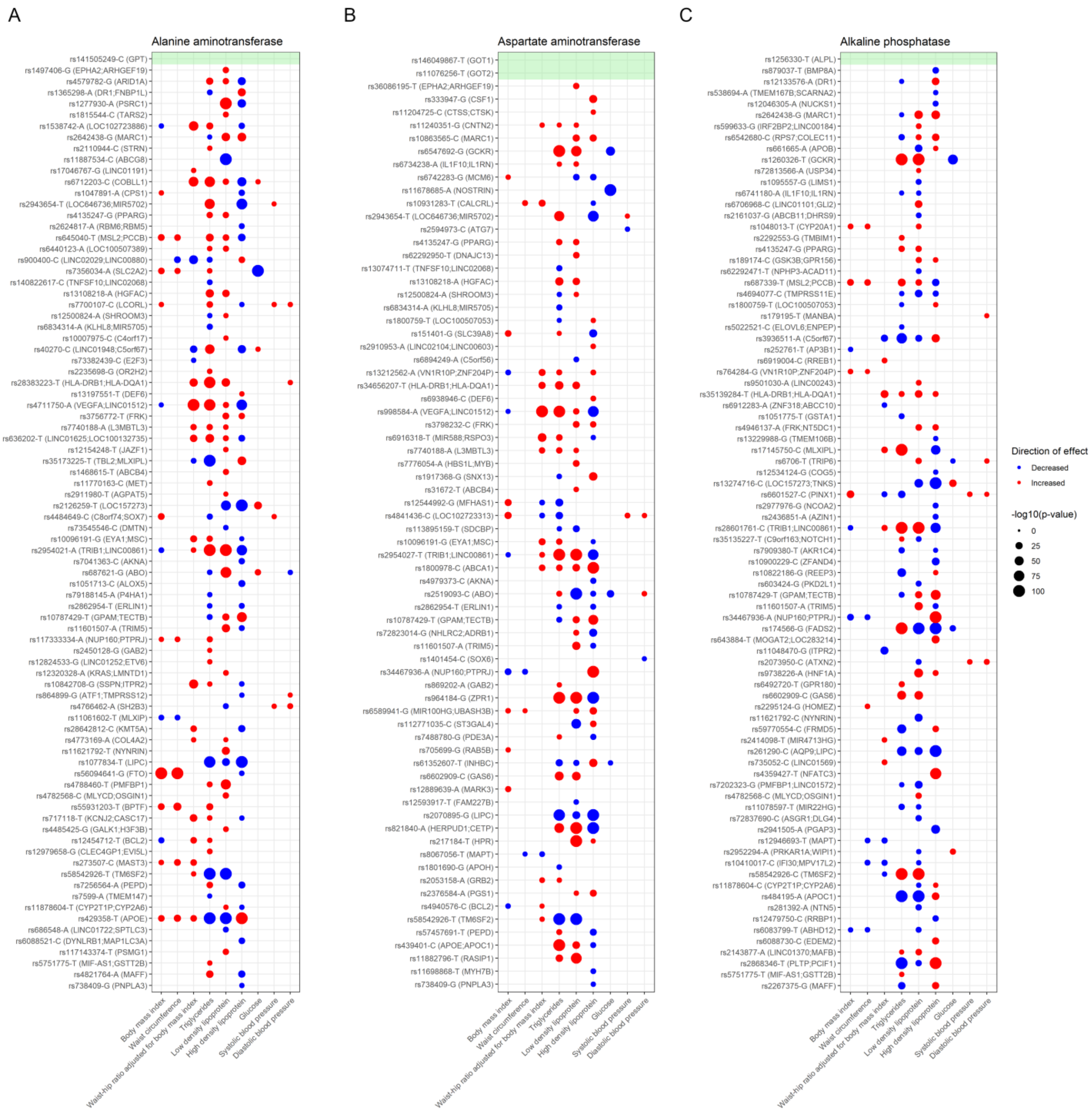

(A-C) Associations between metabolic traits and variants increasing (A) alanine aminotransferase (ALT), (B) aspartate aminotransferase (AST), or (C) alkaline phosphatase (ALP). For all panels, only genome wide-significant associations ($p < 5 \times 10^{-8}$) are shown. Red indicates that the liver enzyme-increasing allele increases the trait, while blue indicates that it decreases it. Larger circles indicate lower p value. Data are from genome-wide association studies in UK BioBank. Green highlighting indicates variants that affect the liver enzymes themselves.

Supplementary Figure 3: Effects of liver enzyme-increasing variants on serum/plasma metabolites.

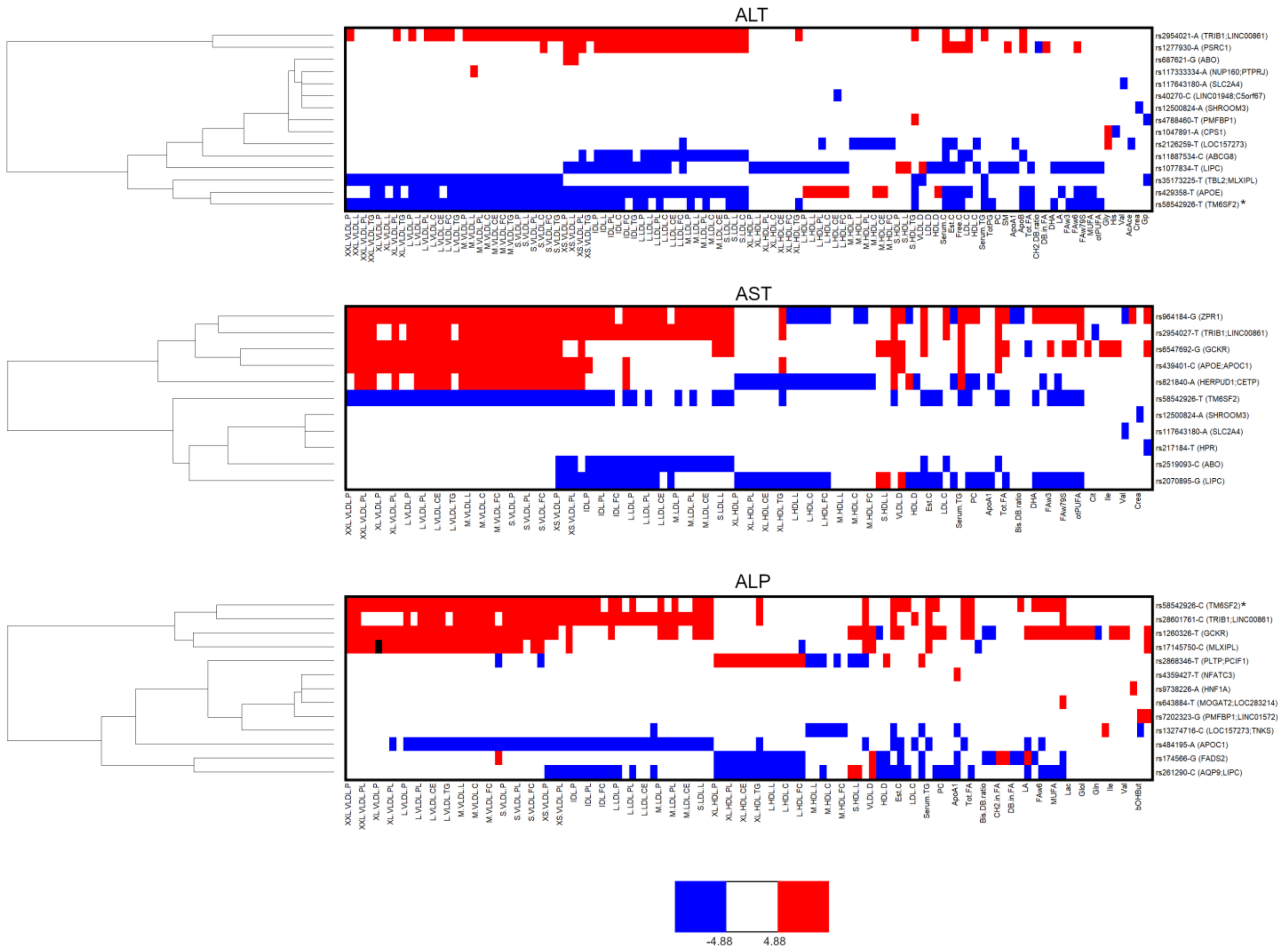

Associations between variants associated with alanine aminotransferase (ALT), aspartate aminotransferase (AST), or alkaline phosphatase (ALP) and serum/plasma metabolites. Data on associations between genetic variants and serum/plasma metabolite concentrations are from [17]. Red indicates that the liver enzyme-increasing allele increases metabolite concentration, blue that it decreases it, and white that there is no significant association. A Bonferroni correction for 123 metabolites and 378 genetic variants ($p < 1.1 \times 10^{-6}$ or $|Z| > 4.88$) was used. Hierarchical clustering of genetic variants was performed using Z scores for variant-metabolite associations as a distance matric. * rs58542926-C (*TM6SF2*) had opposite directions on ALT and ALP.

Supplementary Figure 4: Associations between aspartate aminotransferase polygenic risk score and cirrhosis and steatosis.

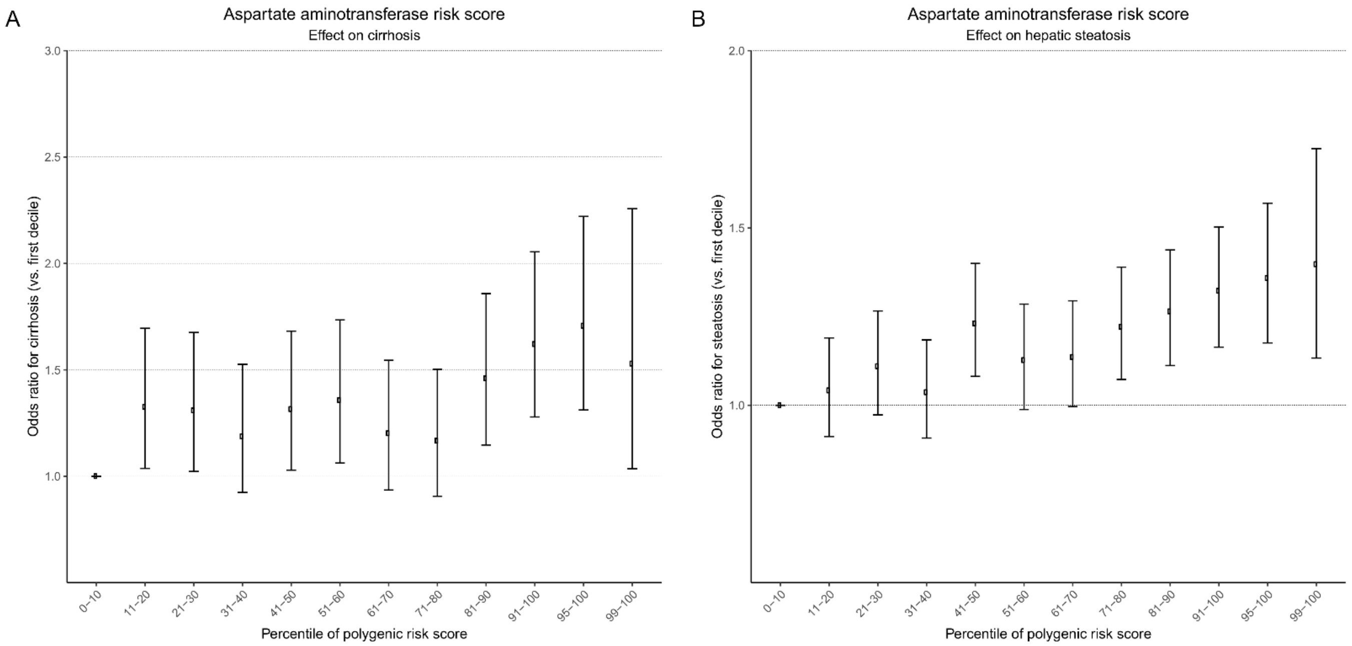

(A-B) Association between percentile of aspartate aminotransferase polygenic risk score on (A) cirrhosis or (B) steatosis. All results are depicted as odds ratios for cirrhosis or steatosis relative to individuals in the 0-10th percentile of polygenic risk score, adjusted for sex, age, age^2, and principal components 1-10.

Supplementary References

1 Berge, K. E. *et al.* Accumulation of dietary cholesterol in sitosterolemia caused by mutations in adjacent ABC transporters. *Science (New York, N.Y.)* **290**, 1771-1775, doi:10.1126/science.290.5497.1771 (2000).

2 Solca, C. *et al.* Sitosterolaemia in Switzerland: molecular genetics links the US Amish-Mennonites to their European roots. *Clinical genetics* **68**, 174-178, doi:10.1111/j.1399-0004.2005.00472.x (2005).

3 Buch, S. *et al.* A genome-wide association scan identifies the hepatic cholesterol transporter ABCG8 as a susceptibility factor for human gallstone disease. *Nature genetics* **39**, 995-999, doi:10.1038/ng2101 (2007).

4 Chong, J. X., Ouwenga, R., Anderson, R. L., Waggoner, D. J. & Ober, C. A population-based study of autosomal-recessive disease-causing mutations in a founder population. *American journal of human genetics* **91**, 608-620, doi:10.1016/j.ajhg.2012.08.007 (2012).

5 Rees, D. C. *et al.* Stomatocytic haemolysis and macrothrombocytopenia (Mediterranean stomatocytosis/macrothrombocytopenia) is the haematological presentation of phytosterolaemia. *British journal of haematology* **130**, 297-309, doi:10.1111/j.1365-2141.2005.05599.x (2005).

6 Mannucci, L. *et al.* Beta-sitosterolaemia: a new nonsense mutation in the ABCG5 gene. *European journal of clinical investigation* **37**, 997-1000, doi:10.1111/j.1365-2362.2007.01880.x (2007).

7 Rios, J., Stein, E., Shendure, J., Hobbs, H. H. & Cohen, J. C. Identification by whole-genome resequencing of gene defect responsible for severe hypercholesterolemia. *Human molecular genetics* **19**, 4313-4318, doi:10.1093/hmg/ddq352 (2010).

8 Tsutsumi, S. *et al.* The novel gene encoding a putative transmembrane protein is mutated in gnathodiaphyseal dysplasia (GDD). *American journal of human genetics* **74**, 1255-1261, doi:10.1086/421527 (2004).

9 Marconi, C. *et al.* A novel missense mutation in ANO5/TMEM16E is causative for gnathodiaphyseal dyplasia in a large Italian pedigree. *European journal of human genetics : EJHG* **21**, 613-619, doi:10.1038/ejhg.2012.224 (2013).

10 Bolduc, V. *et al.* Recessive mutations in the putative calcium-activated chloride channel Anoctamin 5 cause proximal LGMD2L and distal MMD3 muscular dystrophies. *American journal of human genetics* **86**, 213-221, doi:10.1016/j.ajhg.2009.12.013 (2010).

11 Finckh, U. *et al.* Prenatal diagnosis of carbamoyl phosphate synthetase I deficiency by identification of a missense mutation in CPS1. *Human mutation* **12**, 206-211, doi:10.1002/(sici)1098-1004(1998)12:3<206::aid-humu8>3.0.co;2-e (1998).

12 Aoshima, T. *et al.* Novel mutations (H337R and 238-362del) in the CPS1 gene cause carbamoyl phosphate synthetase I deficiency. *Human heredity* **52**, 99-101, doi:10.1159/000053360 (2001).

13 Kurokawa, K. *et al.* Molecular and clinical analyses of Japanese patients with carbamoylphosphate synthetase 1 (CPS1) deficiency. *Journal of human genetics* **52**, 349-354, doi:10.1007/s10038-007-0122-9 (2007).

14 Klaus, V. *et al.* Highly variable clinical phenotype of carbamylphosphate synthetase 1 deficiency in one family: an effect of allelic variation in gene expression? *Clinical genetics* **76**, 263-269, doi:10.1111/j.1399-0004.2009.01216.x (2009).

15 Hu, L. *et al.* Recurrence of carbamoyl phosphate synthetase 1 (CPS1) deficiency in Turkish patients: characterization of a founder mutation by use of recombinant CPS1 from insect cells expression. *Molecular genetics and metabolism* **113**, 267-273, doi:10.1016/j.ymgme.2014.09.014 (2014).

16 Hamada, T. *et al.* Lipoid proteinosis maps to 1q21 and is caused by mutations in the extracellular matrix protein 1 gene (ECM1). *Human molecular genetics* **11**, 833-840, doi:10.1093/hmg/11.7.833 (2002).

17 Novarino, G. *et al.* Exome sequencing links corticospinal motor neuron disease to common neurodegenerative disorders. *Science (New York, N.Y.)* **343**, 506-511, doi:10.1126/science.1247363 (2014).

18 Santos-Cortez, R. L. *et al.* Autosomal-Recessive Hearing Impairment Due to Rare Missense Variants within S1PR2. *American journal of human genetics* **98**, 331-338, doi:10.1016/j.ajhg.2015.12.004 (2016).

19 Siintola, E. *et al.* Cathepsin D deficiency underlies congenital human neuronal ceroid-lipofuscinosis. *Brain : a journal of neurology* **129**, 1438-1445, doi:10.1093/brain/awl107 (2006).

20 Steinfeld, R. *et al.* Cathepsin D deficiency is associated with a human neurodegenerative disorder. *American journal of human genetics* **78**, 988-998, doi:10.1086/504159 (2006).

21 Hersheson, J. *et al.* Cathepsin D deficiency causes juvenile-onset ataxia and distinctive muscle pathology. *Neurology* **83**, 1873-1875, doi:10.1212/wnl.0000000000000981 (2014).

22 Miki, Y. *et al.* A strong candidate for the breast and ovarian cancer susceptibility gene BRCA1. *Science (New York, N.Y.)* **266**, 66-71, doi:10.1126/science.7545954 (1994).

23 Castilla, L. H. *et al.* Mutations in the BRCA1 gene in families with early-onset breast and ovarian cancer. *Nature genetics* **8**, 387-391, doi:10.1038/ng1294-387 (1994).

24 Simard, J. *et al.* Common origins of BRCA1 mutations in Canadian breast and ovarian cancer families. *Nature genetics* **8**, 392-398, doi:10.1038/ng1294-392 (1994).

25 Friedman, L. S. *et al.* Confirmation of BRCA1 by analysis of germline mutations linked to breast and ovarian cancer in ten families. *Nature genetics* **8**, 399-404, doi:10.1038/ng1294-399 (1994).

26 Gayther, S. A. *et al.* Frequently occurring germ-line mutations of the BRCA1 gene in ovarian cancer families from Russia. *American journal of human genetics* **60**, 1239-1242 (1997).

27 Liede, A. *et al.* A breast cancer patient of Scottish descent with germ-line mutations in BRCA1 and BRCA2. *American journal of human genetics* **62**, 1543-1544, doi:10.1086/301889 (1998).

28 Janezic, S. A. *et al.* Germline BRCA1 alterations in a population-based series of ovarian cancer cases. *Human molecular genetics* **8**, 889-897, doi:10.1093/hmg/8.5.889 (1999).

29 Tesoriero, A. *et al.* De novo BRCA1 mutation in a patient with breast cancer and an inherited BRCA2 mutation. *American journal of human genetics* **65**, 567-569, doi:10.1086/302503 (1999).

30 Mefford, H. C. *et al.* Evidence for a BRCA1 founder mutation in families of West African ancestry. *American journal of human genetics* **65**, 575-578, doi:10.1086/302511 (1999).

31 Dorum, A., Heimdal, K., Hovig, E., Inganas, M. & Moller, P. Penetrances of BRCA1 1675delA and 1135insA with respect to breast cancer and ovarian cancer. *American journal of human genetics* **65**, 671-679, doi:10.1086/302530 (1999).

32 Gorski, B. *et al.* Founder mutations in the BRCA1 gene in Polish families with breast-ovarian cancer. *American journal of human genetics* **66**, 1963-1968, doi:10.1086/302922 (2000).

33 Sarantaus, L. *et al.* Multiple founder effects and geographical clustering of BRCA1 and BRCA2 families in Finland. *European journal of human genetics : EJHG* **8**, 757-763, doi:10.1038/sj.ejhg.5200529 (2000).

34 Vallon-Christersson, J. *et al.* Functional analysis of BRCA1 C-terminal missense mutations identified in breast and ovarian cancer families. *Human molecular genetics* **10**, 353-360, doi:10.1093/hmg/10.4.353 (2001).

35 Bergman, A. *et al.* The western Swedish BRCA1 founder mutation 3171ins5; a 3.7 cM conserved haplotype of today is a reminiscence of a 1500-year-old mutation. *European journal of human genetics : EJHG* **9**, 787-793, doi:10.1038/sj.ejhg.5200704 (2001).

36 Vega, A. *et al.* Analysis of BRCA1 and BRCA2 in breast and breast/ovarian cancer families shows population substructure in the Iberian peninsula. *Annals of human genetics* **66**, 29-36, doi:10.1017/s0003480001001014 (2002).

37 Tischkowitz, M. *et al.* Pathogenicity of the BRCA1 missense variant M1775K is determined by the disruption of the BRCT phosphopeptide-binding pocket: a multi-modal approach. *European journal of human genetics : EJHG* **16**, 820-832, doi:10.1038/ejhg.2008.13 (2008).

38 Domchek, S. M. *et al.* Biallelic deleterious BRCA1 mutations in a woman with early-onset ovarian cancer. *Cancer discovery* **3**, 399-405, doi:10.1158/2159-8290.cd-12-0421 (2013).

39 Sawyer, S. L. *et al.* Biallelic mutations in BRCA1 cause a new Fanconi anemia subtype. *Cancer discovery* **5**, 135-142, doi:10.1158/2159-8290.cd-14-1156 (2015).

40 Freire, B. L. *et al.* Homozygous loss of function BRCA1 variant causing a Fanconi-anemia-like phenotype, a clinical report and review of previous patients. *European journal of medical genetics* **61**, 130-133, doi:10.1016/j.ejmg.2017.11.003 (2018).

41 Seo, A. *et al.* Mechanism for survival of homozygous nonsense mutations in the tumor suppressor gene BRCA1. *Proceedings of the National Academy of Sciences of the United States of America* **115**, 5241-5246, doi:10.1073/pnas.1801796115 (2018).

42 Gal, A. *et al.* Mutations in MERTK, the human orthologue of the RCS rat retinal dystrophy gene, cause retinitis pigmentosa. *Nature genetics* **26**, 270-271, doi:10.1038/81555 (2000).

43 Ebermann, I. *et al.* Truncating mutation of the DFNB59 gene causes cochlear hearing impairment and central vestibular dysfunction. *Human mutation* **28**, 571-577, doi:10.1002/humu.20478 (2007).

44 Ksantini, M., Lafont, E., Bocquet, B., Meunier, I. & Hamel, C. P. Homozygous mutation in MERTK causes severe autosomal recessive retinitis pigmentosa. *European journal of ophthalmology* **22**, 647-653, doi:10.5301/ejo.5000096 (2012).

45 Jurkat-Rott, K. *et al.* A calcium channel mutation causing hypokalemic periodic paralysis. *Human molecular genetics* **3**, 1415-1419, doi:10.1093/hmg/3.8.1415 (1994).

46 Ptacek, L. J. *et al.* Dihydropyridine receptor mutations cause hypokalemic periodic paralysis. *Cell* **77**, 863-868, doi:10.1016/0092-8674(94)90135-x (1994).

47 Chabrier, S., Monnier, N. & Lunardi, J. Early onset of hypokalaemic periodic paralysis caused by a novel mutation of the CACNA1S gene. *Journal of medical genetics* **45**, 686-688, doi:10.1136/jmg.2008.059766 (2008).

48 Ke, T., Gomez, C. R., Mateus, H. E., Castano, J. A. & Wang, Q. K. Novel CACNA1S mutation causes autosomal dominant hypokalemic periodic paralysis in a South American family. *Journal of human genetics* **54**, 660-664, doi:10.1038/jhg.2009.92 (2009).

49 Kung, A. W., Lau, K. S., Fong, G. C. & Chan, V. Association of novel single nucleotide polymorphisms in the calcium channel alpha 1 subunit gene (Ca(v)1.1) and thyrotoxic periodic paralysis. *The Journal of clinical endocrinology and metabolism* **89**, 1340-1345, doi:10.1210/jc.2003-030924 (2004).

50 Yoneda, Y. *et al.* De novo and inherited mutations in COL4A2, encoding the type IV collagen alpha2 chain cause porencephaly. *American journal of human genetics* **90**, 86-90, doi:10.1016/j.ajhg.2011.11.016 (2012).

51 Ruiz-Perez, V. L. *et al.* Mutations in a new gene in Ellis-van Creveld syndrome and Weyers acrodental dysostosis. *Nature genetics* **24**, 283-286, doi:10.1038/73508 (2000).

52 Ye, X. *et al.* A novel heterozygous deletion in the EVC2 gene causes Weyers acrofacial dysostosis. *Human genetics* **119**, 199-205, doi:10.1007/s00439-005-0129-2 (2006).

53 Shen, W., Han, D., Zhang, J., Zhao, H. & Feng, H. Two novel heterozygous mutations of EVC2 cause a mild phenotype of Ellis-van Creveld syndrome in a Chinese family. *American journal of medical genetics. Part A* **155a**, 2131-2136, doi:10.1002/ajmg.a.34125 (2011).

54 D'Asdia, M. C. *et al.* Novel and recurrent EVC and EVC2 mutations in Ellis-van Creveld syndrome and Weyers acrofacial dyostosis. *European journal of medical genetics* **56**, 80-87, doi:10.1016/j.ejmg.2012.11.005 (2013).

55 Keller, M. D. *et al.* Mutation in IRF2BP2 is responsible for a familial form of common variable immunodeficiency disorder. *The Journal of allergy and clinical immunology* **138**, 544-550.e544, doi:10.1016/j.jaci.2016.01.018 (2016).

56 Lopez-Herrera, G. *et al.* Deleterious mutations in LRBA are associated with a syndrome of immune deficiency and autoimmunity. *American journal of human genetics* **90**, 986-1001, doi:10.1016/j.ajhg.2012.04.015 (2012).

57 Alangari, A. *et al.* LPS-responsive beige-like anchor (LRBA) gene mutation in a family with inflammatory bowel disease and combined immunodeficiency. *The Journal of allergy and clinical immunology* **130**, 481-488.e482, doi:10.1016/j.jaci.2012.05.043 (2012).

58 Charbonnier, L. M. *et al.* Regulatory T-cell deficiency and immune dysregulation, polyendocrinopathy, enteropathy, X-linked-like disorder caused by loss-of-function mutations in LRBA. *The Journal of allergy and clinical immunology* **135**, 217-227, doi:10.1016/j.jaci.2014.10.019 (2015).

59 Pinz, H. *et al.* De novo variants in Myelin regulatory factor (MYRF) as candidates of a new syndrome of cardiac and urogenital anomalies. *American journal of medical genetics. Part A* **176**, 969-972, doi:10.1002/ajmg.a.38620 (2018).

60 Chitayat, D. *et al.* An Additional Individual with a De Novo Variant in Myelin Regulatory Factor (MYRF) with Cardiac and Urogenital Anomalies: Further Proof of Causality: Comments on the article by Pinz et al. (). *American journal of medical genetics. Part A* **176**, 2041-2043, doi:10.1002/ajmg.a.40360 (2018).

61 Qi, H. *et al.* De novo variants in congenital diaphragmatic hernia identify MYRF as a new syndrome and reveal genetic overlaps with other developmental disorders. *PLoS genetics* **14**, e1007822, doi:10.1371/journal.pgen.1007822 (2018).

62 Wang, K., Zhou, B., Kuo, Y. M., Zemansky, J. & Gitschier, J. A novel member of a zinc transporter family is defective in acrodermatitis enteropathica. *American journal of human genetics* **71**, 66-73, doi:10.1086/341125 (2002).

63 Kury, S. *et al.* Identification of SLC39A4, a gene involved in acrodermatitis enteropathica. *Nature genetics* **31**, 239-240, doi:10.1038/ng913 (2002).

64 Nakano, A., Nakano, H., Nomura, K., Toyomaki, Y. & Hanada, K. Novel SLC39A4 mutations in acrodermatitis enteropathica. *The Journal of investigative dermatology* **120**, 963-966, doi:10.1046/j.1523-1747.2003.12243.x (2003).

65 Saleheen, D. *et al.* Human knockouts and phenotypic analysis in a cohort with a high rate of consanguinity. *Nature* **544**, 235-239, doi:10.1038/nature22034 (2017).

66 Diodato, D. *et al.* VARS2 and TARS2 mutations in patients with mitochondrial encephalomyopathies. *Human mutation* **35**, 983-989, doi:10.1002/humu.22590 (2014).

67 Taylor, R. W. *et al.* Use of whole-exome sequencing to determine the genetic basis of multiple mitochondrial respiratory chain complex deficiencies. *Jama* **312**, 68-77, doi:10.1001/jama.2014.7184 (2014).
